# Co-dependent formation of the *Toxoplasma gondii* subpellicular microtubules and inner membrane skeleton

**DOI:** 10.1128/mbio.01389-25

**Published:** 2025-08-13

**Authors:** Klemens Engelberg, Ciara Bauwens, David J. P. Ferguson, Marc-Jan Gubbels

**Affiliations:** 1 Department of Biology, Boston College 6019, Chestnut Hill, Massachusetts, USA; 2 Department of Biological and Medical Sciences, Oxford Brookes University, and NDCLS, Oxford University 6395, Oxford, United Kingdom; UT Southwestern Medical Center, Dallas, Texas, USA

**Keywords:** microtubules, *Toxoplasma*, *Sarcocystis*, γ-tubulin, SFA, APR, γTuRC, IMC, alveoli

## Abstract

One of the defining features of apicomplexan parasites is their cytoskeleton composed of alveolar vesicles, known as the inner membrane complex (IMC) undergirded by an intermediate filament-like protein network and an array of subpellicular microtubules (SPMTs). In *Toxoplasma gondii*, this specialized cytoskeleton is involved in all aspects of the disease-causing lytic cycle and notably acts as a scaffold for parasite offspring in the internal budding process. Despite advances in our understanding of the architecture and molecular composition, insights pertaining to the coordinated assembly of the scaffold are still largely elusive. Here, *T. gondii* tachyzoites were dissected by advanced, iterative expansion microscopy (pan-expansion microscopy), revealing new insights into the very early sequential formation steps of the tubulin and IMC scaffold. A comparative study of the related parasite *Sarcocystis neurona* revealed that different MT bundling organizations of the nascent SPMTs correlate with the number of central and basal alveolar vesicles. In the absence of a so-far identified MT nucleation mechanism, we genetically dissected *T. gondii* γ-tubulin and γ-tubulin complex proteins 4, 5, and 6 (GCP4/5/6). While γ-tubulin depletion abolished the formation of the tubulin scaffold, a set of MTs still formed that suggests SPMTs are nucleated at the outer core of the centrosome. Depletion of GCP4/5/6 interfered with the correct assembly of nascent SPMTs into the forming daughter buds, further indicating that the parasite utilizes the γ-tubulin ring complex in tubulin scaffold formation.

**IMPORTANCE:**

Apicomplexan protozoan parasites rely on their specialized cytoskeleton to form offspring. The cytoskeleton serves as an essential scaffold for the emerging daughter cells and is formed by the inner membrane complex (IMC) and underlying subpellicular microtubules (SPMTs). In *Toxoplasma gondii*, the IMC is composed of several membranous sacks and supported by 22 SPMTs, the latter are evenly spaced around the apical end of mature parasites. Although many advances have been made, little is known about the earliest steps of scaffold formation. Here, we gain unprecedented insights into IMC and SPMT establishment via iterative expansion microscopy and comparative cell biology. We show that at the onset of division, SPMTs are grouped and reveal that the number of groups determines the number of IMC sacks that are assembled. We further dissect the parasite’s γ-tubulin ring complex and show that it is critically involved in scaffold formation.

## INTRODUCTION

The phylum Apicomplexa is largely comprised of obligate intracellular parasites with a defining apical constellation of secretory organelles and cytoskeletal structures uniquely evolved to facilitate invasion of another host cell. The assembly of these apical elements is orchestrated by the centrosome and unfolds through a budding process wherein the cortical cytoskeleton serves as a scaffold for *de novo* assembly of secretory organelles (1). Although there is significant customization of the parasite size and budding mode to each parasitic niche, the assembly of the cortical cytoskeleton is the principal driver of budding and is thus conserved across the phylum (1–3). The cortical cytoskeleton is not only important for cell division (budding) but is also responsible for gliding motility required for successful host cell invasion (4). Hence, it is pivotal for the success of apicomplexan parasites.

The cortical cytoskeleton is categorized as an epiplastin-based membrane skeleton, which is principally different from the actin-spectrin-based cytoskeleton present in mammalian cells (5). The three main elements of the parasite’s cytoskeleton are a set of flattened alveolar vesicles (alveoli), upheld by a set of intermediate filament-like proteins (named alveolins in general, but in Apicomplexa known as alveolin-domain-containing inner membrane complex [IMC] proteins) (6–9), and subpellicular microtubules (SPMTs) anchored by the apical polar ring (APR) at the apical end of the parasite (10, 11). The number of SPMTs and alveolar plates is highly variable across parasite species and developmental stages within the same species (2). The apical end of the cytoskeleton is characterized by the conoid, with various elaboration degrees in different parasites, composed of unconventional tubulin fibers and two polar rings (12).

The tachyzoite stage of *Toxoplasma gondii* is an established model apicomplexan in which many of the cytoskeleton and cell division details have been unraveled (1, 4, 6). In the asexual stages of *T. gondii* and related coccidian parasites like *Neospora caninum* and *Sarcocystis neurona*, the number of SPMTs is 22, while the number of alveolar plates varies (2, 13–15). The IMC (alveoli together with alveolin-domain-containing IMC proteins) rests on the SPMTs that radiate from the APR (10) and are evenly spaced in the mature parasite.

**Fig 1:**
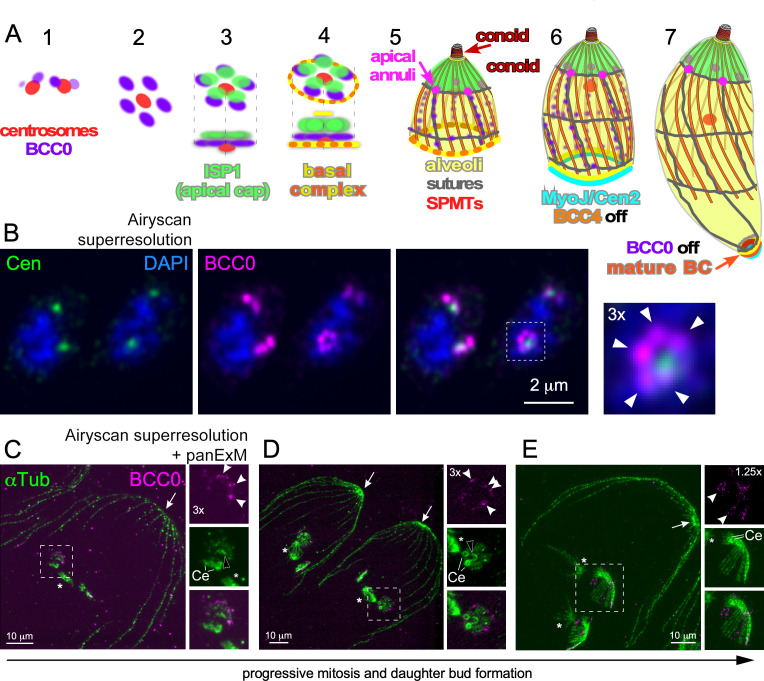
Early daughter cytoskeleton assembly displays fivefold symmetry, alternating between nascent SPMT (nSPMT) rafts and the foundation of longitudinal alveolar sutures. (A) Schematic representation of *T. gondii* cytoskeleton assembly. Stages 1–7 represent advancing steps in daughter cytoskeleton scaffold assembly. Modified from reference 21. (B) Original observation of BCC0 bud initiation as five distinct puncta, with fivefold symmetry (puncta marked by arrowheads). Cen, *Hs*Cen2 antiserum highlighting the centrosome; 4´,6-diamidino-2-phenylindole (DAPI): DNA. BCC0: BCC0-5xV5. (C through E) Pan-expansion microscopy (pan-ExM) of three different parasites progressing through mitosis and early bud assembly. Asterisks mark mitotic spindle; arrows mark the apical end of the mother’s cytoskeleton (conoid); open arrowheads the nascent conoid of daughter buds; arrowheads mark the BCC0-5xV5 signals deposited before nSPMT assembly in five foci around the centrioles (Ce), which subsequently accumulates between the nSPMTs rafts. αTub, α-tubulin antiserum (mAb 12G10).

The *T. gondii* tachyzoite divides by arguably the least complex mode of apicomplexan cell division, endodyogeny, forming two new daughter parasites per one round of division within the cytosol of the mother cell (1, 6, 16). Each daughter buds from a bipartite centrosome that is divided in the inner core, which controls the nuclear cycle, and the outer core, controlling bud assembly (17, 18). Bud formation is dependent on two proteins homologous to algal striated fiber assemblin (SFA) that form a fiber positioning and anchoring the bud scaffold to the centrosome (16, 18–20). Bud formation is initiated by scaffolding and signaling proteins, which are initially laid out in a five- or sometimes sixfold symmetry (21–26) (Fig. 1A). The daughter buds grow first bidirectionally to later adapt to a strictly apical to basal growth trajectory (16, 21, 27). All three elements of the cortical cytoskeleton are intertwined and appear to assemble in parallel rather than sequentially (21, 28, 29). However, the understanding of early bud formation and especially how the assembly of the three elements is coordinated is limited. Concomitant with the APR and SPMTs, the conoid is formed, consisting of 14 unconventional tubulin fibers (30) and two intra-conoidal microtubules (ICMTs) (31). With the progression of division, the dividing nucleus is encapsulated by the forming daughter buds (mitosis and cytokinesis progress in parallel) (32). At the basal end of the forming daughter buds lies the functional equivalent of the contractile cytokinetic ring, the basal complex (BC), which is responsible for yoking the (+)-ends of the SPMTs and later on for tapering and constricting the nascent daughters (21, 33–37). Division concludes with disassembly of the mother’s IMC followed by the daughter parasites emerging while depositing their mother’s plasma membrane on their IMC (38, 39).

How the SPMTs are nucleated is still not known in detail, but the APR has been proposed as an unusual microtubule organizing center (MTOC) in which the (−)-ends of SPMTs are inserted between blunt projections to form a cogwheel-like pattern (10, 40–42). This assignment is supported by its close apposition to the SPMTs and conoid as well as the presence of a centrosomal SAS6-like protein in the APR (the centrosome harbors its own SAS6) (43). This insight, together with several other suggestions and the key role for an SFA fiber, indicates that daughter budding either has shared ancestry or at least co-opted elements and mechanisms shared with cilium formation in eukaryotic cells (44). Several proteins have been identified that facilitate stability of the APR (kinesin A, APR1, RNG2, and AC9 and 10), but none directly drive bud initiation (22, 42, 45, 46). Stability of SPMTs is modulated by several previously identified proteins, but similar to the APR, for none a convincing role in bud initiation has been established (28, 41, 47–50).

γ-Tubulin and its associated complex (γ-TuC) is a known nucleator of MTs (51), and γ-TuC key components (γ-tubulin, GCP 2, 3, and 4) have been identified in the *T. gondii* genome (52). γ-Tubulin has previously been localized to the outer core of the centrosome (18). Its molecular function, however, has not yet been established. Given the close proximity between the centrosomal outer core and the forming daughter bud, we hypothesized that γ-TuC may be functionally involved in the assembly of the tubulin scaffold.

We have recently adopted an advanced iterative expansion microscopy (ExM) technique, called pan-ExM (53). Pan-ExM relies on two consecutive rounds of sample expansion, which allowed us to physically increase parasite size by up to a factor of 13. Using this new technology, we revealed the organization of the 22 nascent SPMTs (nSPMTs) in five distinct “rafts” around the APR at bud initiation. We compared this organization to *S. neurona* merozoites, which put down 11 pairs of two SPMTs, an organization that mirrors twice the number of alveolar sheets (14). Our data further show that not all nSPMTs form at the same time, but rather are sequentially assembled on the APR, indicating a distinct area of nucleation. When we examined the localization of γ-tubulin by pan-ExM, we observed it at the spindle poles, the centrioles, and, unexpectedly, also at the nascent APR (nAPR), specifically at the onset of division. Rapid depletion of γ-tubulin abolishes the formation of the tubulin scaffold and results in paired SPMTs emanating from centrioles. Thus, γ-tubulin localization and function highlight it as a pivotal component of daughter cell formation. Functional dissection of the SFA component SFA2 and a set of γ-tubulin complex proteins further allowed us to gain insights into the assembly hierarchy of the daughter scaffold.

Overall, the combination of advanced imaging and genetic dissection provides deep new insights into how SPMT and alveolar architecture are coordinated to form the template for daughter bud formation.

## RESULTS

### Fivefold symmetry of the *T. gondii* tachyzoite early daughter scaffold

We recently reported that the *T. gondii* daughter-specific protein BCC0 assembles in a distinct fivefold symmetry around the duplicated centrosomes early in division. Upon progression of budding, BCC0 is deposited in longitudinal stripes, reminiscent of the daughter IMC sutures (Fig. 1A and B) (21). To refine our understanding of these very early steps in daughter cell assembly, we adopted pan-expansion microscopy (pan-ExM) (53–55), which in our hands results in an approximately 13-fold increase of sample size. Co-staining of α-tubulin and BCC0 through early daughter budding using pan-ExM revealed that BCC0 localizes in between distinct SPMT “rafts,” composed of four individual SPMTs, which are formed around the centrioles (Fig. 1C through E). Temporally, templating of the SPMTs starts while the metaphase mitotic spindle is present (Fig. 1C). When the spindle poles separate during anaphase (Fig. 1D), the organization of SPMTs in rafts of four nSPMTs, as described recently by U-ExM (56), is still visible, but the nascent scaffold transitions from a flat plane into a cone-shaped conformation (Fig. 1E). BCC0 remained visible between SPMT rafts with continued growth of the nascent buds (Fig. S1A), which is suggestive of coordination between BCC0 and SPMT formation.

**Fig 2:**
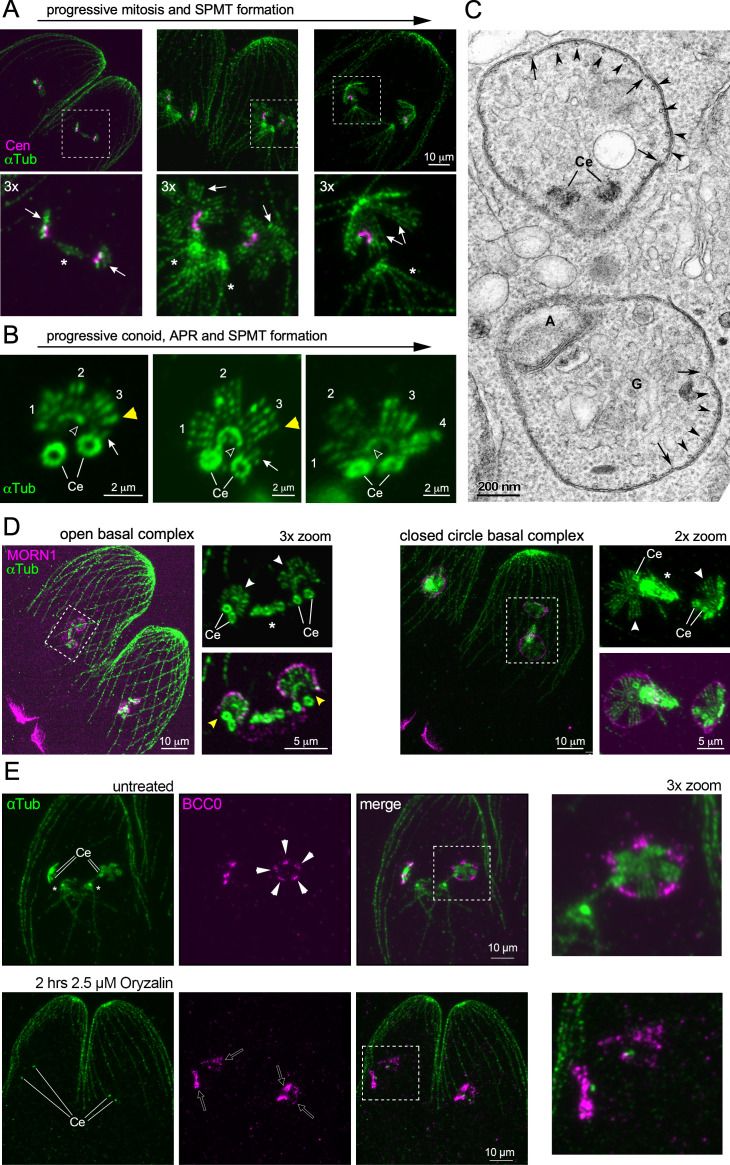
The nSPMTs display a fivefold symmetrical organization early in the division, in connection with the IMC and the basal complex. (A) Pan-ExM of early budding parasites co-stained with *Hs*Centrin2 (Cen) and α-tubulin (αTub: mAb 12G10) reveals that the centrioles are connected by a centrin fiber. SPMT formation starts during mitotic anaphase (left panel; mitotic spindle between the centrosomes). Fivefold symmetrical rafts of nSPMTs assemble during telophase (middle and right panels), which transitions from a flat organization (middle panel) into a domed orientation (right panel). Arrows: nSPMT rafts; asterisks: mitotic spindles. (B) Pan-ExM snapshots of sequential stages of conoid (open arrowheads) and nSPMT formation. nSPMT rafts are numbered. Arrows mark pairs of short MTs that are being added to the growing scaffold. Note that the ICMT pair is visible within the conoid in the middle panel. Ce, centrioles. (C) Transmission electron microscopy of early daughter budding. Cross-section through daughter buds wherein nSPMT rafts are visible with IMC membrane sheets on top. Arrowheads mark individual nSPMTs; arrows mark breaks in the membrane sheets of the nascent IMC alveoli. A: apicoplast, Ce: centriole; G: Golgi apparatus. (D) Pan-ExM of the MT cytoskeleton co-stained with MORN1 antiserum, highlighting how the nascent basal complex interfaces with the early scaffold (nSPTMs). Ce, centrioles; asterisks mark mitotic spindle; white arrowheads mark the nSPMT rafts; yellow arrowheads mark the discontinuity of the nascent basal complex assembled close to the (+)-end of nSPMTs. (E) Pan-ExM reveals the differential susceptibility of microtubules to oryzalin treatment. As previously observed (58, 59), division is not halted by this treatment, but cytoskeleton organization is substantially altered. After oryzalin treatment, BCC0-5xV5 appears as “plaques” (open arrows) around (single) centrioles, contrary to its localization in discrete five foci (arrowheads) between nSPMT rafts in untreated controls. Ce, centrioles; asterisks: mitotic spindle.

Next, we delved into the earliest steps in daughter scaffold formation, as pan-ExM permits viewing these early steps without visual perturbation from the maternal cortical cytoskeleton. Upon staining the MTs with α-tubulin, pan-ExM clearly resolves the two centrioles in the *T. gondii* centrosome, which are marked with centrosome marker *Hs*Cen2 forming a fiber between the centrioles (21) (Fig. 2A). Also, α-tubulin is present in the mitotic spindle and highlights the nSPMTs. In line with recent studies (56, 57), the 22 nSPMTs are organized in five to six distinct rafts of propeller-like symmetry, consisting of four rafts (each with four nSPMTs) plus one raft with six nSPMTs (Fig. 2A, middle panel) or five rafts of four SPMTs plus a single raft of two SPMTs (Fig. S1B, untreated), with the fivefold assembly reported as the most abundantly observed one (56). We noted that in the earliest stage shown in Fig. 2A, the nSPMTs are very few in number and very short. Subsequently, we looked for earlier steps in nSPMT formation and found several cases of three rafts (each with four nSPMTs) + two individual nSPMTs and four rafts (each with four nSPMTs) assembled around the forming conoid (Fig. 2B, arrowhead). At this stage, the nascent scaffold is reminiscent of a horseshoe and is on one side in close proximity to one of the centrioles (Fig. 2B). This horseshoe was recently assigned to be the early conoid (56). We frequently observed a pair of two short MTs, overlaying with a centriole (arrows in Fig. 2B, left and center panels), and others apparently being added to one side of the forming scaffold (Fig. 2B, yellow arrowhead). Taken together, we interpret these data into a model wherein the nSPTMs grow in pairs of two and are subsequently organized into rafts of four nSPMTs, whereas two additional nSPMTs are either on their own or added to another nSPMT raft to make a total of six. Germane to the early organization of nSPMTs, this suggests a defined area of nucleation and a symmetrical coordination with BCC0 laying down the IMC. This subsequently outlines the pattern for the growing cytoskeleton, i.e., the position of the apical annuli and alveolar sheets (e.g., Fig. 1A).

### SPMTs assembly pattern provides foundation for alveoli plate architecture

The fivefold symmetrical nSPMT organization in Fig. 1C correlates with the five distinct foci, previously detected for BCC0. To directly visualize the membrane of the IMC, we performed transmission electron microscopy (TEM). Indeed, we observed four nSPMTs undergirding membrane sheets with gaps between them (Fig. 2C). From this, we inferred that BCC0 localizes to the gaps as it becomes imbedded in the sutures between the five and six alveolar plates of the two rows of central and one row of basal alveoli (21). In addition, the TEM showed a centriole pair in close apposition to one side of the early daughter bud, mirroring our pan-ExM observations in Fig. 2B.

Another early and crucial component of daughter buds is the scaffolding protein MORN1, which assembles the BC at the basal end of the cytoskeleton scaffold (21, 33, 60, 61). During the early stages while the nSPMTs are still forming, we observed MORN1 as a horseshoe-shaped open circle around the (+)-ends of the nSPMTs (Fig. 2D, left panels). Once all nSPMTs are formed, MORN1 transitioned into a closed circle configuration of the mature BC (Fig. 2D, right panels). However, at this point, the nSPMTs are still organized in rafts, indicating the even spreading of SPMTs around the nascent nAPR must occur at a later stage and is independent of BC circle closure.

To further probe the correlation between nSPMTs and the alveolar architecture, we chemically perturbed SPMT formation using the MT destabilizer oryzalin (58, 59). We then assessed IMC formation using either BCC0 (Fig. 2E) or the early marker IMC32, a membrane-anchored protein essential for daughter bud initiation displaying the same early symmetry as BCC0 (23) (Fig. S1B). In parasites treated for 2 h with oryzalin, we observed single centrioles that are far separated from each other, indicating that, surprisingly, centrosome splitting progressed normally despite the lack of centriole duplication (Fig. 2E). Centrosomal splitting indicated that budding had been initiated, although the spindle and nSPMTs were absent. BCC0 and IMC32, however, still accumulated around the single centrioles but did not display the typical symmetry seen in the untreated controls (Fig. 2E; Fig. S1B) (23). Collectively, these observations support a model wherein the nSPMT organization defines the symmetry of the alveolar membrane organization.

### Comparative biology of nSPMTs and alveolar architecture

**Fig 3:**
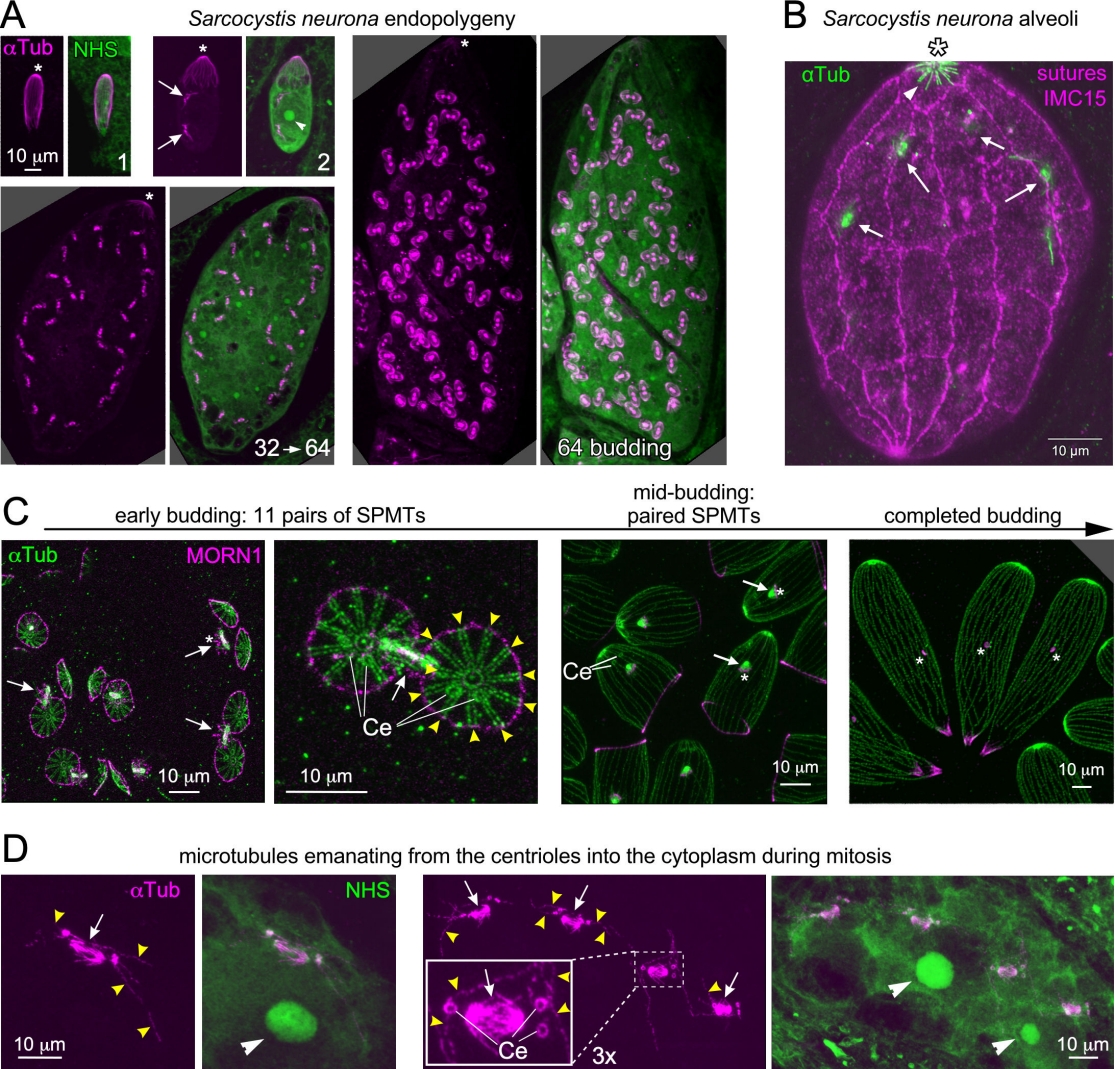
Spindle and SPMTs in *Sarcocystis neurona* endopolygeny. (A) Representative stages of endopolygeny imaged by single expansion. 1: a just invaded, single merozoite; 2: early schizont undergoing the first mitosis; 32→64: late schizont going through the last round of mitosis (32 spindles) coupled to budding 64 merozoites—the first outlines of the nSPMTs are visible at the ends of each spindle; 64 budding: later budding schizont with the nSPMTs well defined. Asterisks mark the apical end (conoid) of the mother’s cytoskeleton, which is visible until the very end; arrows mark mitotic spindles; arrowhead marks the nucleolus. All panels are from the same expansion experiment and are shown on the same scale. αTub: α-tubulin antiserum (mAb 12G10). NHS: protein density highlighted with NHS-ester conjugated to ATTO488. (B) Single expansion imaging of the *S. neurona* IMC. The IMC is composed of an apical cap and 3 × 11 alveolar sheets (note that only the front of the schizont is displayed here). The sutures between the sheets are marked by stable overexpression of YFP-TgIMC15 (63). Note the SPMTs (arrowhead) only extend along the apical cap in this mid-stage schizont. Arrows mark the mitotic spindles. αTub: α-tubulin antiserum (mAb 12G10). See Fig. S2 for a wider z-range of this schizont. (C) Pan-ExM of *S. neurona* daughter budding stages co-stained with α-tubulin (αTub) and MORN1 antiserum. α-Tubulin marks the spindle, centrioles, and SPMTs. MORN1 highlights the centrocone and the basal complex. Ce: centriole; arrows mark mitotic spindle; yellow arrowheads mark the 11 pairs of nSPMTs; asterisks: centrocone. (D) Pan-ExM of *S. neurona* schizonts, zoomed in on the spindles. MTs of unknown identity emanate from the centrioles into the cytoplasm during mitosis. Two different parasites are shown: left panels undergoing the first round of mitosis (two spindles); right panels undergoing the third round of mitosis (eight spindles). Ce: centriole; arrows mark overlapping mitotic spindles; yellow arrowheads mark the MTs of unknown identity; white arrowheads mark the nucleolus. αTub: α-tubulin antiserum (mAb 12G10). NHS: protein density highlighted with NHS-ester conjugated to ATTO488.

To further cement the connection between nSPMTs and the IMC alveolar sheet architecture, we turned to the closely related coccidian parasite *Sarcocystis neurona. S. neurona* is the causative agent of equine protozoal myoencephalitis (62). Unlike *T. gondii* tachyzoites, *S. neurona* merozoites replicate by endopolygeny, where the parasite undergoes several rounds of S-phase and mitosis without completing karyokinesis, followed by a final round of S- and M-phase coupled to daughter budding, resulting in 64 emerging merozoites (Fig. 3A) (1). In relation to the question at hand, *S. neurona* merozoites, like *T. gondii* tachyzoites, harbor 22 SPMTs, but have twice as many alveolar sheets: 11 total (visualized in Fig. 3B using the IMC suture marker TgIMC15-YFP [63]) vs 5-6 for *T. gondii* tachyzoites (15). Hence, we anticipated the nSPMT to reflect the increased number of alveoli. This is indeed what we observed early in daughter cytoskeleton assembly where nSPMTs formed 11 pairs, each consisting of two nSPMTs. Like in *T. gondii*, the BC marker MORN1 is located close to the (+)-end of SPMTs during development and highlights the basal end of the forming bud. The observed MT “pairing” was maintained through the mid-budding stage until budding was completed and the merozoites matured (Fig. 3C). Interestingly, however, the SPMTs in mature merozoites were alternating between an SPMT about two-thirds the length of the cell and another SPMT that extended all the way to the end of the merozoite, connecting to the basal complex (Fig. 3C, right panel). This contrasts with *T. gondii* tachyzoites, where none of the SPMTs reach the very basal end and only extend to 60% of the length of the mature tachyzoite, although some variation in SPMT length is seen here as well (14).

Other distinctive observations on the *S. neurona* cortical cytoskeleton highlight the modest apical cap in schizonts, which is the only place where the mother’s SPMTs are seen (Fig. 3B). In fact, during the progression of endopolygeny, it appears that the mother’s SPMTs are shrinking (Fig. 3A: all panels are on the same scale and from the same expansion experiment). This implies a putative level of catastrophe on the SPMTs (+)-ends, which goes against the current dogma of extreme SPMT stability (49). Lastly, we very frequently observed MTs emanating from centrioles into the cytoplasm present at mitotic spindles in schizonts before the final round of S/M paired with daughter budding. Notably, bundled spindle MTs were present at the same time, but these were all clearly oriented toward the opposite centriole pair (Fig. 3D; Fig. S2). These cytoplasmically oriented MTs were somewhat reminiscent of astral MTs seen in higher eukaryotes that function in orienting the mitotic spindle within the cell. Alternatively, this could be a leaky assembly of nSPMTs during mitotic rounds uncoupled from budding.

Overall, the comparative analysis of *S. neurona* as a parasite closely related to *T. gondii* highlighted several subtly distinctive features. Most notably, the paired nSPMT organization located at bud initiation corresponds directly with the number of alveolar membrane plates. Together, this suggests an architectural layout rule, at least in the coccidia, where nSPMT raft organization directs alveolar plate organization.

### γ-Tubulin is critical for correct spindle and nSPMT assembly in *T. gondii*

**Fig 4:**
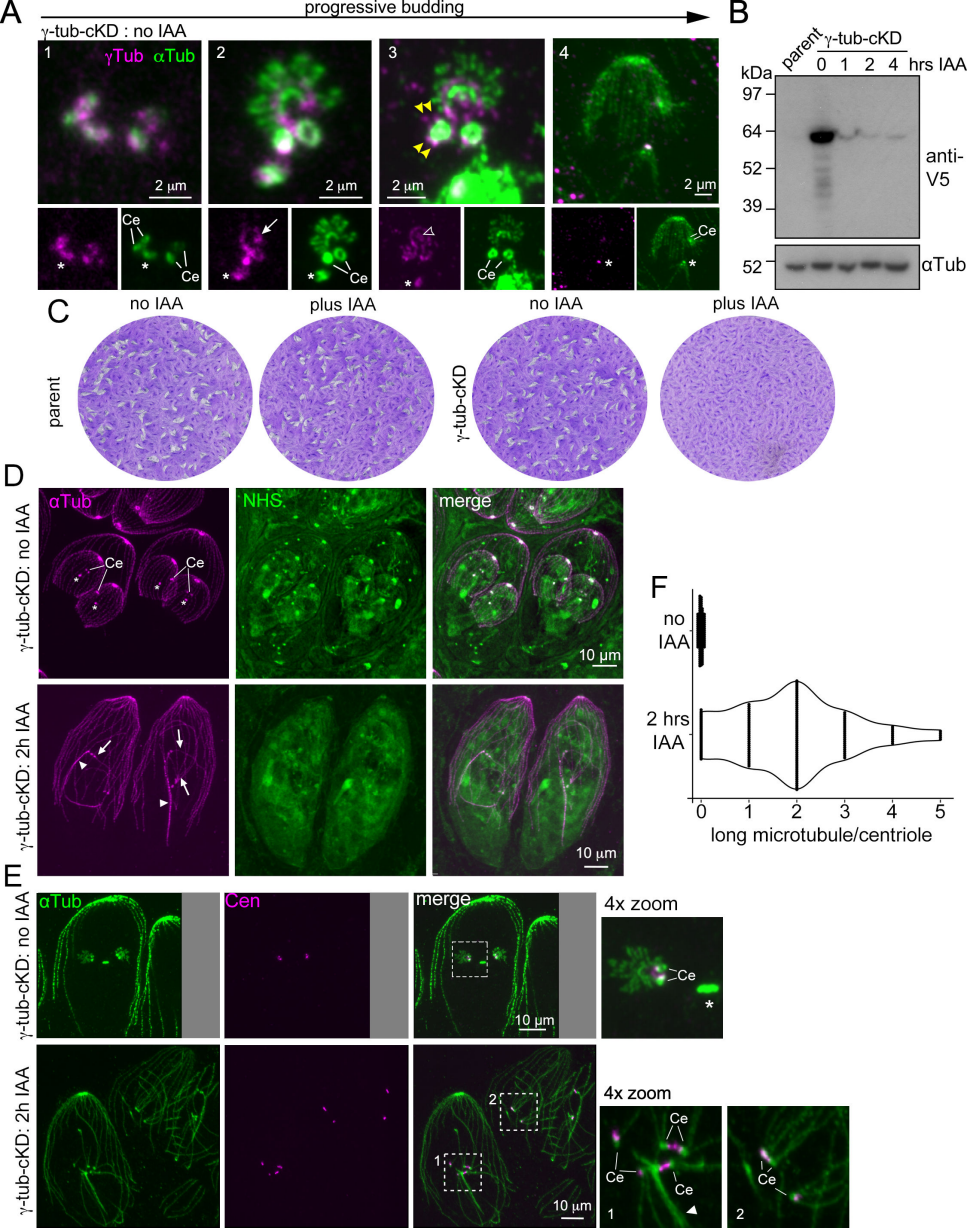
γ-Tubulin is essential for cell division. (A) Pan-ExM of parasites expressing γ-tubulin endogenously tagged at the C-terminus with mAID-5xV5 (γ-tub-cKD), co-stained with V5 and α-tubulin antisera. Asterisks: the spindle or spindle pole; arrow: horseshoe shape at the nAPR; open arrowhead: γ-tubulin at the ICMT; yellow arrowhead: γ-tubulin pairs of different length protruding from the centriole (Ce). (B) Kinetics of γ-tub-cKD depletion upon indole-3-acetic acid (IAA) addition visualized by western blot. The mAID system allows fast protein degradation. Parent line is RH∆Ku80-Tir1. αTub: α-tubulin antiserum (mAb 12G10) is used as loading control. (C) Seven-day plaque assay of γ-tubulin depletion demonstrates its essentiality for the lytic cycle. Parent line is RH∆Ku80-Tir1. (D) Pan-ExM of γ-tubulin-cKD parasites ± IAA, stained for α-tubulin (αTub) and protein density with NHS-ester conjugated to ATTO488. In IAA-treated parasites, the mother MT populations appear normal, whereas two daughter MT populations are visible: one (arrows) connected to round structures (putatively centrioles), and the other (arrowheads) presents as a long bundle of MTs. Ce: pairs of centrioles; asterisks: spindle poles. (E) Pan-ExM of γ-tubulin-cKD parasites ± IAA, co-stained for α-tubulin and *Hs*Centrin2 (Cen: centrosomes). A variable number of unstructured microtubules emanates from typically one side of each centriole, which are connected by Centrin. Numbered boxes are expanded in panels on the right. Ce: centrioles; asterisks: spindle poles; arrowhead: long bundle of Mts. (F) Quantification of panel D for the number of long MTs emanating from centrioles. The majority of centrioles in IAA-treated parasites, roughly 30%, is connected to two MTs; the remainder is a mixture of zero to five MTs. For each condition, 39 mature parasites were quantified with 156 centrioles for no IAA and 145 centrioles for 2 h IAA.

Having established the early organization of nSPMTs, we next asked how the SPMTs are nucleated. To this end, we examined the subcellular localization of γ-tubulin by pan-ExM in *T. gondii*. Using conventional microscopy, γ-tubulin was localized to the outer core of the centrosomes, which is where the centrioles reside (18). We endogenously tagged the C-terminus of γ-tubulin with the mini auxin-inducible degron (mAID) coupled to five V5 epitopes (γ-tub-cKD) (Fig. S3A and B). At the onset of mitosis, before budding starts, we observed γ-tubulin on and around the centrioles as well as the early mitotic spindle (Fig. 4A, panel 1). When the nSPMTs started forming, γ-tubulin resided on the spindle pole, the centrioles, and in a horseshoe shape between the forming conoid and the (−)-ends of the nSPMTs (Fig. 4A, panel 2). γ-Tubulin therefore seems to highlight the nAPR at bud initiation. We also detected γ-tubulin at the ICMTs within the conoid (Fig. 4A, panel 3, open arrow). During subsequent anaphase, when nSPMT rafts are fully formed, γ-tubulin is still present at the spindle pole and the centrioles, though with lower abundance, while it is absent from the apical end of the forming scaffold (Fig. 4A, panel 4). Thus, γ-tubulin is associated with the formation of all MT populations in tachyzoites. Furthermore, we sometimes see pairs of short γ-tubulin pairs emanating from a centriole (Fig. 4A, panel 3, yellow arrowheads). We speculate that these could be the basis of new SPMT pairs being initiated close to the centriole before they are transferred onto the nAPR.

To functionally dissect γ-tubulin, we took advantage of the mAID tag facilitating effective depletion of most γ-tubulin within hours of indole-3-acetic acid (IAA) treatment (64) (Fig. 4B; Fig. S3C and D). When treated for 7 days with IAA, γ-tubulin-depleted parasites lost plaque-forming capacity, demonstrating that it is essential to complete the lytic cycle (Fig. 4C). Next, we assessed the effect of γ-tubulin depletion on the first round of daughter cell formation (2 h IAA), with a special focus on the MT cytoskeleton. The maternal SPMTs and conoid appeared unperturbed, but structures associated with daughter cell formation were severely disrupted. We observed two distinct MT populations, with one consisting of individual MTs connected to centrioles, the other formed by a bundle of MTs (Fig. 4D and E; Fig. S3D). Centriole duplication and separation was not affected by γ-tubulin depletion, as indicated by co-staining with *Hs*Cen2 (Fig. 4E), but the centrioles appeared denser, without their usually detected donut-like shape. We enumerated the variable number “centriolar” MTs, which ranged from 0 to 5, with two MTs being the most commonly observed number (Fig. 4F).

To test for consequences of γ-tubulin depletion beyond 2 h, we performed a 24 h IAA treatment experiment, upon which the SPMTs of the mother are also perturbed (Fig. S4A). During this period, parasites failed to generate a new round of daughter scaffolds (Fig. S4A, tubulin staining); however, the substantial size increase of the mother parasites suggested that many organelles still duplicated. We tested this by focusing on Golgi-endosomal-related compartments and the apicoplast and found in virtually all parasites enlarged organelles, with the apicoplast failing to segregate (Fig. S4B and C). Enlarged nuclei, as judged by 4´,6-diamidino-2-phenylindole (DAPI) staining, further suggested that absence of γ-tubulin affects nuclei division (Fig. s4B and C, arrows).

**Fig 5:**
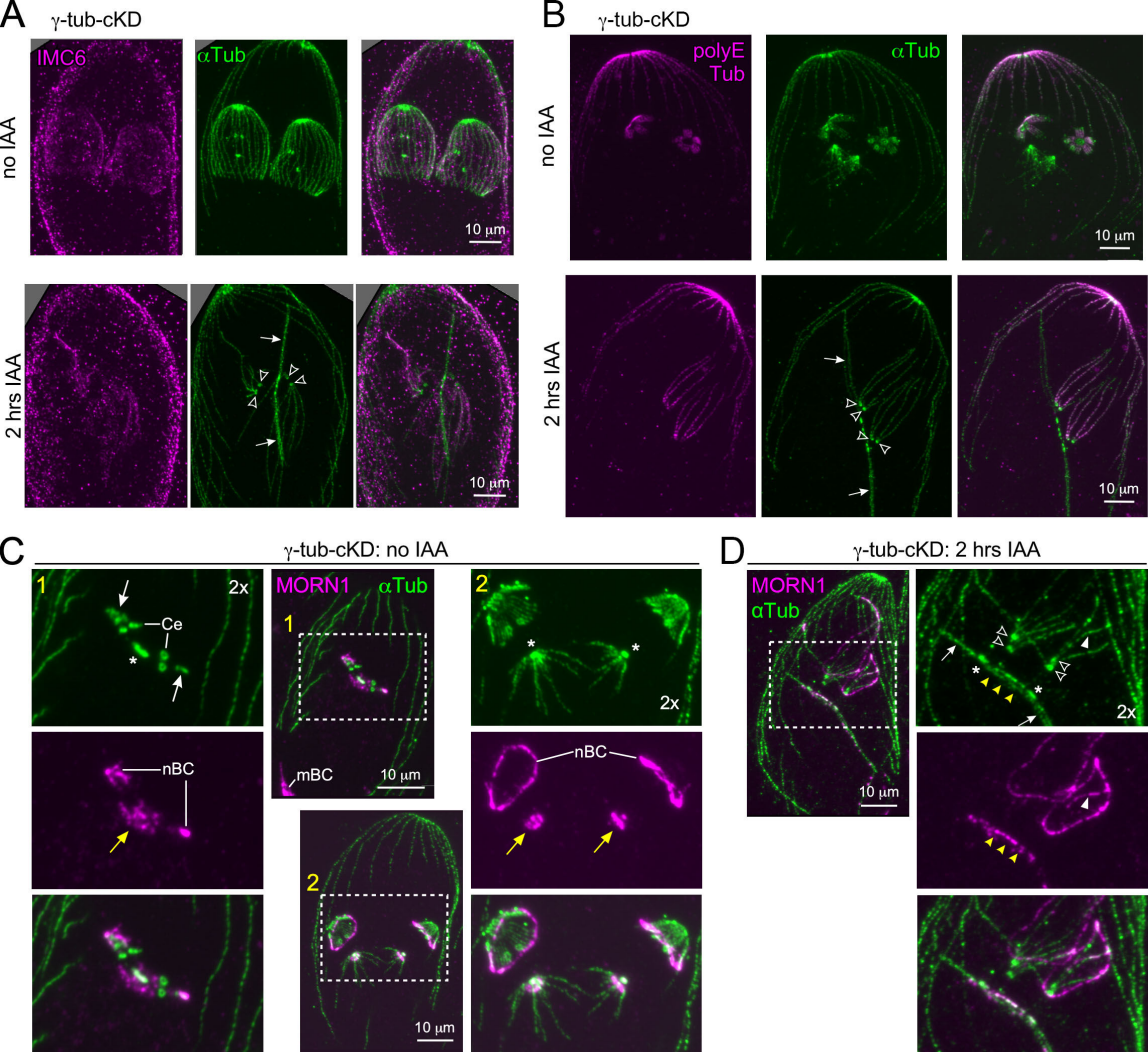
Identity of microtubule populations in γ-tubulin-depleted parasites. (A) Pan-ExM of control and 2 h γ-tubulin-depleted parasites stained with α-tubulin and IMC6 antisera. Arrows: bundled MT, not coated with IMC6; open arrowheads: centrioles from which IMC6-coated MTs are emanating. (B) Pan-ExM of control and 2 h γ-tubulin-depleted parasites stained for α-tubulin and polyE tubulin antisera. Arrows: bundled MT, not marked with polyglutamylated tubulin; open arrowheads: centrioles from which polyglutamylated tubulin-coated MTs are emanating. (C, D) Pan-ExM of control and 2 h γ-tubulin-depleted parasites stained with α-tubulin (αTub, mAb12G10) and MORN1 antisera. (C) Yellow-1-labeled panels show a very early budding cytoskeleton, with only a few nSPMTs (arrow) of the scaffold surrounded by the nBCs. MORN1 also highlights the centrocone (yellow arrow) in the nuclear envelope, where the spindle reaches into the nucleus. Yellow-2-labeled panels show early budding parasites with dome-shaped nSPMTs coated with the nBC close to the nSPMT (+)-ends. Yellow arrows: centrocones. Dotted-line boxed areas are twofold enlarged. Yellow numbers indicate corresponding panels. mBC, mother parasite’s BC. (D) After γ-tubulin depletion (2 h IAA), MORN1 localizes in a semi-circle to the “centriolar” MTs and partially coats them (white arrowheads). MORN1 also localizes to the middle of the bundled MT, highlighting the centrocone. Asterisk: mitotic spindle; open arrowheads: centrioles; yellow arrowheads: bundled MTs coated with MORN1, marking the mitotic spindle embedded in the nuclear envelope. Dotted-line boxed areas are twofold enlarged.

To identify the observed MT populations and to assess the fate of the spindle and the nSPMTs in γ-tubulin-depleted parasites, we stained with the IMC marker IMC6 (27, 65) and the SPMT marker polyglutamylated (PolyE) tubulin (22, 66). This revealed that the long MTs emanating from centrioles were associated with the IMC and decorated with PolyE (Fig. 5A and B). Notably, neither marker labeled the bundled MTs. Furthermore, MORN1—marking, among other structures, the nascent BC (nBC) (Fig. 5C)—was observed forming semi-circular structures surrounding the long “centriolar” MTs following γ-tubulin depletion, suggesting the presence of an incompletely formed BC (Fig. 5D). Additionally, the MORN1 signal appeared to align with a subset of the “centriolar” MTs (Fig. 5D, white arrowheads). Collectively, the analysis of IMC6, PolyE, and MORN1 permitted a firm assignment of the long “centriolar” MTs as nSPMTs.

By extension of the above findings, the bundled MTs most likely represented the spindle MTs, which, however, were much extended in length when compared to wild-type spindles. We confirmed this by use of MORN1. MORN1 is present at the BC as well as the mitotic spindle at the centrocone, which is the structure embedded in the nuclear envelope through which the spindle MT are attached to the kinetochores (Fig. 5C, yellow arrows) (33). More saliently, MORN1 predominantly stained the bundled MTs in the middle after γ-tubulin depletion (Fig. 5D, yellow arrowheads), most likely highlighting the area where the spindle penetrates into the nucleus. The nature of MTs extending beyond the MORN1-stained bundle could either be extended spindle MTs or mis-oriented spindle MTs. Our current data cannot differentiate between these scenarios. In conclusion, γ-tubulin is critical for establishing all MT populations in tachyzoites and, germane to the MT daughter scaffold, facilitates correct nSPMT assemblage.

### Bud integrity is severely affected by γ-tubulin depletion

To further determine the relationship between nSPMTs and the daughter IMC in γ-tubulin-depleted parasites, we explored daughter bud assembly using the central and basal IMC markers IMC3 (27) and IMC6 (65) as well as the apical cap marker ISP1 (67). Interestingly, we found no significant difference between the number of parasites that initiated budding in the presence or absence of γ-tubulin, as judged by daughter IMC3 staining (Fig. S5A and B), but the assembly of the daughter IMC is disorganized and results in nearly 100% misshapen buds (Fig. S5A and C).

**Fig 6:**
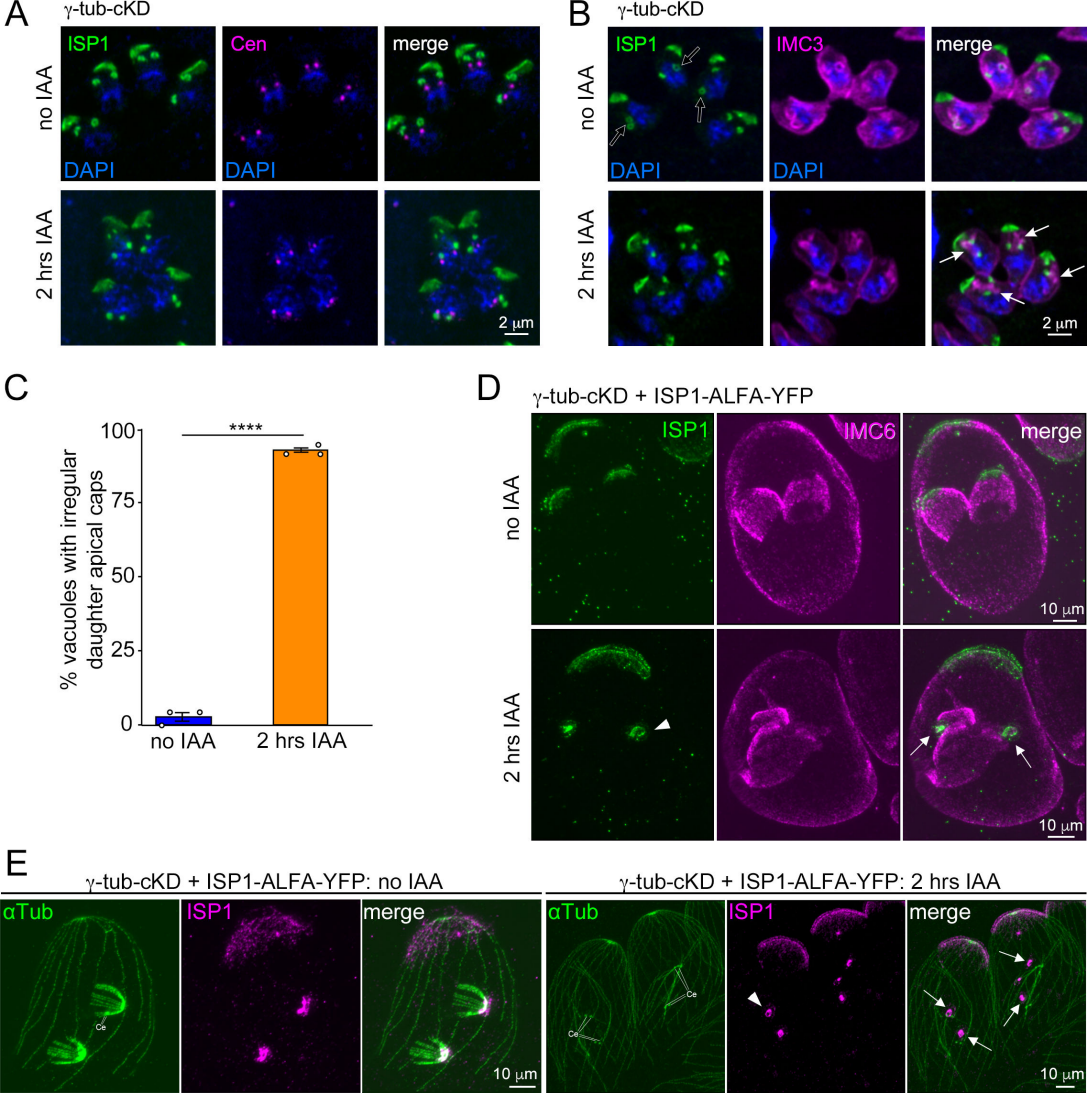
γ-Tubulin depletion prevents correct formation of the apical cap. (A, B) Airyscan superresolution images after 2 h of γ-tubulin depletion using two different co-stains: (A) ISP1 (apical cap) and *Hs*Centrin2 (Cen: centrosomes); (B) ISP1 (apical cap) and IMC3 (mother and daughter IMC). After γ-tubulin depletion, daughter ISP1 appears normally distributed around the centrosomes but fails to be positioned apical to the unstructured daughter IMC. The signal appears more focused (“dot-like”) and is lacking ring conformation, as seen in controls (open arrows), suggesting incompletely formed apical caps. ISP1 and IMC3 were visualized with respective antisera. DAPI highlights DNA. Arrows: unstructured daughter IMC. (C) Quantification of apical cap formation in γ-tub-cKD parasites treated ± IAA. Fifty dividing vacuoles were quantified for each condition in *n* = 3 biological replicates. The appearance of daughter apical caps was analyzed via IMC3 and ISP1 antisera using immunofluorescence assay. Error bars denote SEM. Data points indicate individual replicates. Significance was determined by Welch’s two-tailed *t*-test. ****, *P* < 0.0001. (D) Pan-ExM of γ-tub-cKD parasites, showing incompletely formed daughter apical caps (ISP1) when treated with IAA. ISP1 was endogenously tagged with ALFA-tag-YFP and visualized with ALFA-tag antiserum. Mother and daughter IMC are highlighted by IMC6 antiserum. Arrowhead marks ring-like appearance of ISP1 signal. Arrows indicate ISP1 signal disjoined from unstructured daughter IMC. (E) Pan-ExM of γ-tub-cKD parasites, showing apical cap positioning in relation to the daughter scaffold. ISP1 was endogenously tagged with ALFA-tag-YFP and visualized with GFP antiserum. Arrowhead marks ring-like appearance of ISP1 signal; arrows indicate disconnection from “centriolar” nSPMTs. Ce, centrioles. αTub, α-tubulin antiserum (mAb 12G10).

The localization of daughter apical caps appeared unperturbed upon 2 h γ-tubulin depletion, forming close to the duplicated centrosomes (Fig. 6A). This indicates that this budding event does not require correct assembly of nSPMTs. Upon γ-tubulin depletion, however, ISP1 signal appeared disconnected from the unstructured IMC and more focused (“dot-like”) compared to controls, where ISP1 has a ring conformation at bud initiation (Fig. 6B, open arrows; Fig. 6C). Pan-ExM revealed that ISP1 signal still resembled a ring-like structure after γ-tubulin depletion (Fig. 6D and E, arrowheads); however, the apical cap did not form to the extent seen in controls and appeared disjoined from the unstructured daughter IMC (Fig. 6D, arrows) and from the residual “centriolar” nSPMTs (Fig. 6E, arrows). Taken together, these data consolidate the insights gleaned from the oryzalin experiment (Fig. 2E; Fig. S1B), showing that the MT scaffold—nucleated by γ-tubulin—is essential for the structural integrity of daughter buds, which in turn is critical for correct formation of the IMC.

### The γ-TuRC components are essential scaffolding factors

**Fig 7:**
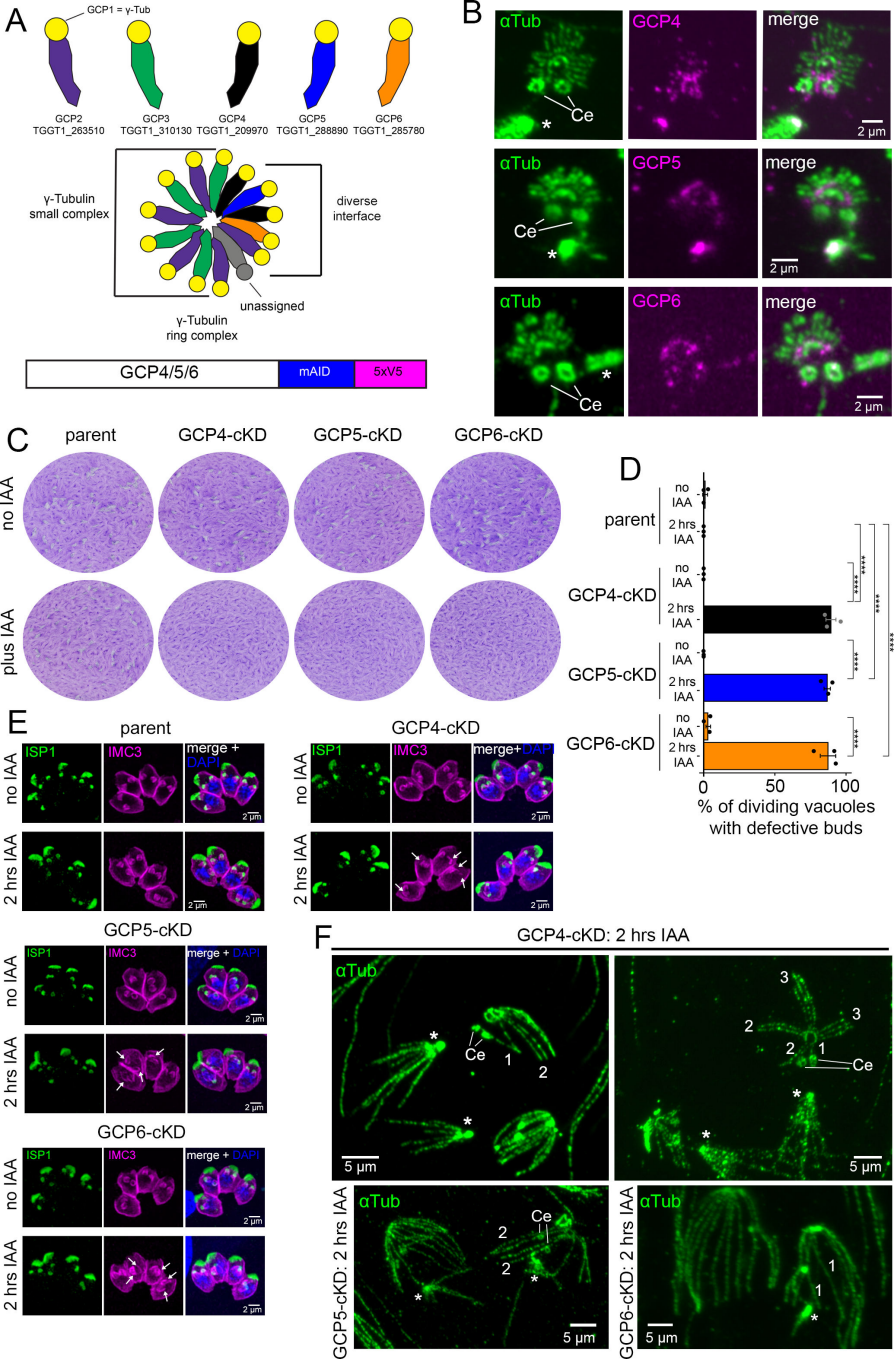
Involvement of γ-TuRC in daughter scaffold formation. (A) *T. gondii* GT1 accession number of GCP2-6. The γ-TuRC “core” is formed by GCP1 (γ-tubulin), GCP2, and GCP3 (γ-tubulin small complex). The diverse interface is formed by the addition of GCP4–6. Model based on human γ-TuRC, adopted from reference 69. Investigation of GCP4/5/6 was done by endogenous 3´ end tagging of the respective gene with mAID and a 5xV5 epitope tag. (B) GCP4/GCP5/GCP6 (GCP4–6-cKD) localize to the spindle poles (asterisks) as well as around the centrioles (Ce), and to the forming scaffold. Imaged by pan-ExM. αTub, α-tubulin antiserum (mAb 12G10). (C) Plaque assays of GCP4/5/6-depleted parasites using the mAID system indicate that GCPs are essential for the lytic cycle. Parent line is RH∆Ku80-Tir1. (D) Quantification of dividing vacuoles that exhibit defective daughter buds ± a 2 h treatment with IAA. Anti-IMC3 and anti-ISP1 antisera were used as markers for daughter bud morphology. *n* = three biological replicates. One-way analysis of variance with Tukey’s honestly significant difference (HSD). ****, *P* < 0.0001. Error bars denote SEM. See Fig. S6B for the percentage share of dividing vacuoles in the examined 100 random vacuoles/replicate. Parent line is RH∆Ku80-Tir1. (E) Representative images of vacuoles, harboring RH∆Ku80-Tir1 (parent) or GCP4/5/6-cKD parasites ± 2 h IAA. Anti-IMC3 indicates IMC, anti-ISP1 the apical cap. DAPI highlights DNA. Arrows indicate defective buds. (F) Pan-ExM of 2 h depletion of GCP4/5/6. Mitosis progresses, and two daughter buds are formed; however, the organization and number of nSPMTs in each raft is irregular (numbers indicate nSPMTs per raft). Ce, centrioles; asterisks: spindle poles. αTub, α-tubulin antiserum (mAb 12G10).

The observed essential involvement of γ-tubulin in scaffold formation led us to further pursue the function of the γ-tubulin ring complex (γ-TuRC). *T. gondii* encodes all six γ-TuRC members ([52]; with GCP5 [TGGT1_288890] and GCP6 [TGGT1_285780] recently being annotated on the ToxoDB genome database [68]) (Fig. 7A). Here, we explored the role of γ-TuRC using conditional GCP4, GCP5, and GCP6-mAID-5xV5 (GCP-cKD) parasite lines (Fig. 7A). When we analyzed the subcellular localization of GCP4-6 via pan-ExM, all proteins were present at the spindle poles, around the centrioles, and at the early scaffold (Fig. 7B). The same localizations were also observed for γ-tubulin (Fig. 4A). Furthermore, similar to γ-tubulin, plaque assays indicated that GCP4-6 are essential for the *T. gondii* lytic cycle (Fig. 7C). After 2 h IAA treatment, we detected GCP depletion (Fig. S6A), which had a significant effect on vacuoles with dividing parasites, where the majority of newly formed daughter buds were defective and did not present the same cone-shaped morphology seen in the parental line or in the absence of IAA (Fig. 7D and E). Analyzed through pan-ExM, the daughter scaffolds were markedly deformed by GCP depletion and exhibited disorganized nSPMTs (Fig. 7F). Here, nSPMT rafts contained less than the usually four nSPMTs but were formed by either one, two, or three nSPMT per raft (as numbered in Fig. 7F). Interestingly, the spindle and the centrioles appeared normal upon GCP depletion under the given condition, although GCP4/5/6 localized to both structures. We then tested the fate of γ-tubulin in GCP-depleted parasites by endogenously tagging γ-tubulin in the GCP6-cKD background. Upon depletion of GCP6, γ-tubulin lost its localization to the forming scaffold, and its abundance at the centrioles and the spindle poles appeared strongly reduced (Fig. S6C, arrows). By western blot, we could, however, confirm that the overall γ-tubulin amount showed only a slight decrease when GCP6 was depleted (Fig. S6D), suggesting that GCP6 depletion disrupts γ-TuRC, which is necessary to facilitate localization of γ-tubulin.

### SFA2 function precedes γ-tubulin actions in bud formation

**Fig 8:**
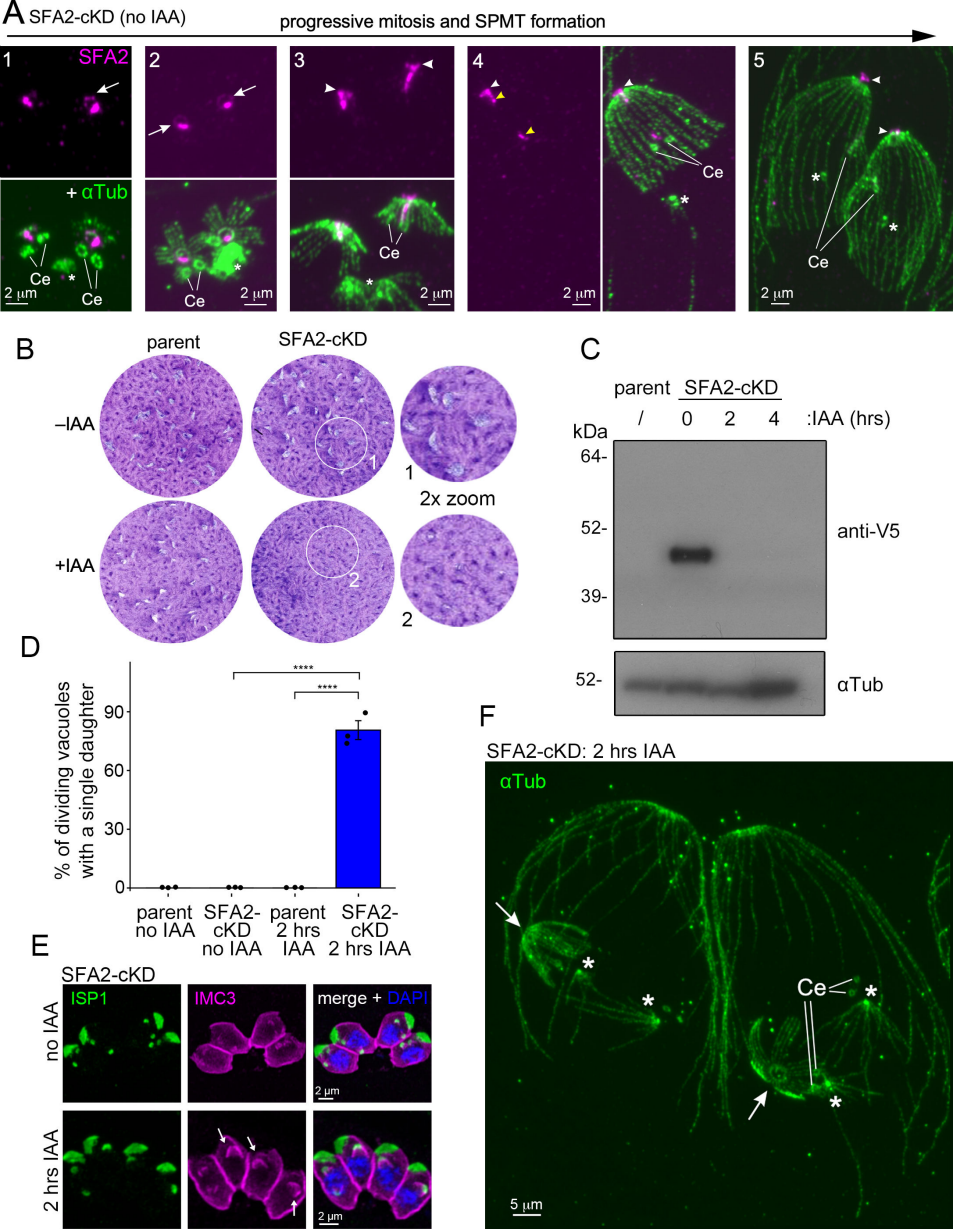
SFA2 depletion impairs bud initiation. (A) SFA2-mAID5xV5 (SFA2-cKD) localization throughout mitosis and early daughter bud assembly Ce, centriole; asterisks: mitotic spindle; arrows: SFA2 ring-like structure aligning with the conoid. Arrowhead: SFA2 signal apical to the daughter conoid; yellow arrowhead marks the broken fiber at mid-budding. Imaged by pan-ExM. αTub, α-tubulin antiserum (mAb 12G10). (B) Seven-day plaque assays of SFA2-depleted parasites using the mAID system. Parent line is RHΔKu80-Tir1. Numbered areas are magnified to show residual plaquing ability of SFA2-cKD parasites in the presence of IAA. Quantification of plaque sizes is shown in Fig. S7A. (C) Western blot of SFA2-cKD parasites treated for 2 or 4 h with IAA. Anti-α-tubulin (αTub: mAb 12G10) is used as a loading control. Parent parasites (RH∆Ku80-Tir1) serve as control for V5 antiserum. (D) Quantification of dividing vacuoles that exhibit only a single daughter bud. One-way analysis of variance with Tukey’s honestly significant difference (HSD). ****, *P* < 0.0001. Error bars denote SEM. *n* = three biological replicates. See Fig. S7B for the percentage share of dividing vacuoles in the examined 100 vacuoles/replicate. Parent line is RH∆Ku80-Tir1. (E) Representative images of dividing SFA2-cKD parasites. Parasites were stained with IMC3 (highlighting the IMC) and ISP1 (highlighting the apical cap) antiserum. DAPI stains DNA. Arrows indicate parasites that only form a single daughter bud. (F) Pan-ExM of 2 h SFA2-depleted parasites. Mitosis progresses normally, but single daughter buds appear at a high frequency. The single buds display a normal nSPMT organization. Ce, centrioles; asterisks: mitotic spindle; arrowheads: apical end of the single daughter bud. αTub, α-tubulin antiserum (mAb 12G10).

The SFA fiber arises from between the centriole pair and extends to the nascent apical complex of the daughter bud (19). The current understanding of its function is positioning the MTOC, which forms the template for the daughter scaffold during cell division (19, 32): SFA’s depletion disrupts daughter scaffold formation completely, assigning it an essential role in the budding process (19). We first re-examined the exact SFA2 localization here by the additional resolution offered by pan-ExM (Fig. 8A). We confirmed the presence of SFA2 between the centriole pair very early in development. In addition, we observed SFA2 in between the conoid and nSPTMs (panels 1 and 2). Once the bud transforms into the cone shape, during metaphase, SFA was seen as a fiber extending all the way from between the centrioles to the apical side of the conoid. Notably, at the conoid, it appears as a flattened disk located above it and the nSPMTs’ (−)-ends (panel 3, arrowhead). Upon further budding, the SFA2 signal became discontinuous (panel 4) and, upon bud maturation, was only retained above the conoid (panel 5). Taken together, SFA2 is present near the nAPR during nSPMT formation but progresses to a localization apical to the nascent scaffold.

Next, we depleted SFA2 using the mAID system, which severely affected parasite proliferation (Fig. 8B) and resulted in a 79% reduction in plaque size (Fig. S7A). A short, 2 h SFA2 depletion resulted in the absence of the protein detected in western blot analyses (Fig. 8C). When dividing vacuoles were examined with IMC3 and ISP1 staining, we found SFA2-depleted parasites to frequently only form a single daughter bud (Fig. 8D and E). By pan-ExM, we found normal appearing centriole pairs and mitotic spindles in dividing parasites, but parasites formed only a single daughter nSPMT scaffold (Fig. 8F), with the nSPMT constellation being remarkably the same as seen in unperturbed parasite buds.

Taken together, we confirm SFA2’s previously reported essential role in establishing the daughter scaffold (19), but our utilization of the mAID system suggests a mechanism that seems to be an all or nothing, i.e., SFA2 depletion below a certain threshold at the centrosome results in no daughter MT scaffold, whereas above this threshold, a normal scaffold is assembled. Notably, in the absence of SFA2, we did not detect any long “centriolar” SPMTs at the scaffold-less centrioles, as seen after γ-tubulin depletion (Fig. 4 and 5), indicating that bud initiation is entirely prevented.

In summary, our data establish a hierarchical order for SFA2 in budding and highlight the essential involvement of γ-tubulin and γ-TuRC in daughter scaffold formation.

## DISCUSSION

**Fig 9:**
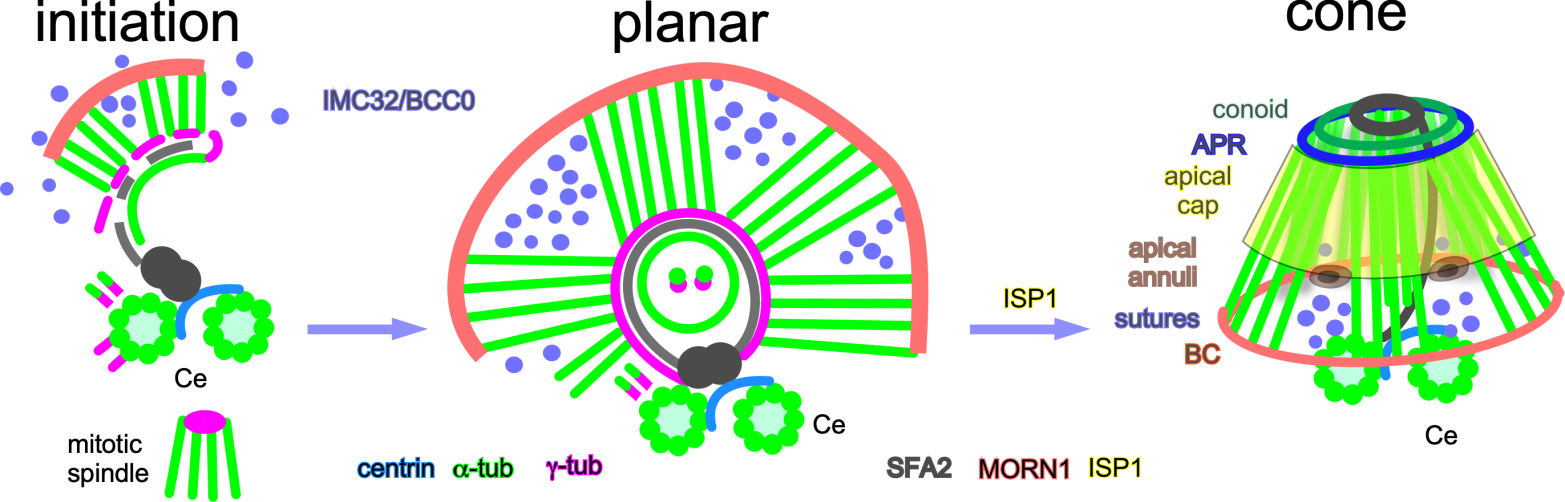
Model of daughter scaffold formation in *T. gondii*. Summarizing schematic: The daughter scaffold is initiated close to the centrioles (Ce) and relies on two factors, γ-tubulin (magenta) for the nucleation and/or stabilization/anchoring of tubulin components, and as previously reported on the SFA fiber (19) (black). The basal complex (MORN1 [33], red), the alveoli (not shown), and IMC components (e.g. BCC0 [21] and IMC32 [23], blue) align with the earliest observable tubulin scaffold structure. SPMTs are nucleated close to the centrioles, presumably in the pericentriolar material of the outer centrosomal core, and subsequently added to the forming APR (highlighted by γ-tubulin/magenta). Closure of the circular conoid and nAPR, in concert with nSPMT addition, establishes the scaffold’s planar configuration. At this point, the basal complex assumes ring shape. Most abundantly, four rafts of four nSPMTs and one raft of six nSPMTs (this study; 56) support the alveoli, which are being added to the growing scaffold (not shown). The apical cap (ISP1 [67], yellow) is formed by a so far incompletely understood process, but presumably establishes the cone shape of the scaffold, which then grows in basal direction. At this point, γ-tubulin no longer localizes to the APR (blue), which is then stabilized by other factors (e.g. KinA/APR1 [42] and/or AC9/10 [22]).

To reconstruct the series of sequential events underlying cell division and how the formation of the different structural components is hierarchically organized, we put our new findings in chronological order, as summarized in Fig. 9. First, we would like to point out a potential caveat in our experimental design: the very short depletion window of 2 h used by us, which is needed to capture the earliest events in the developing phenotypes. Since efficient methods to synchronize *T. gondii* cell division to the degree needed for these experiments have not been established, we cannot be sure that replication did start in the complete absence of any of the proteins we targeted. In addition, the mAID system does not necessarily completely remove all protein, but its extremely fast kinetics of protein depletion make it the best tool available for this study. Either way, putative protein traces can influence the phenotypes we observed.

We define a key role for γ-tubulin, as it is present at the formation site of all the tachyzoite’s MT populations: spindle in the centrosome inner core, centrioles in the centrosome outer core, the nSPMTs surrounding the conoid, and the ICMTs (Fig. 4). Pertinent to daughter scaffold assembly, there are key observations regarding the positioning of γ-tubulin in the extending horseshoe shape emanating from the centriole, which we interpret as the nAPR. As the first sign of the scaffold formation, we observe a short, single MT coated with MORN1 (Fig. 5C, arrows in panel 1). The assembly then progresses by forming nSPMTs in close proximity to the centrioles, as we see paired γ-tubulin (Fig. 4A) as well as α-tubulin appendages sprouting from there (Fig. 2B). These novel paired MT assemblies then apparently transfer from the centrosome to the growing APR, although the mechanics of this step cannot be fully deduced from the images we captured. Supportive data for MT nucleation close to the centrioles are found in *S. neurona* where we observe MTs associated with centrioles as well (Fig. 3D; Fig. S2). Although their function remains unknown for now, their presence suggests that the outer core of the centrosome can nucleate MTs.

In the above model, nSPMT initiation close to the centrioles precedes their transfer to the nAPR. Upon γ-tubulin depletion, we find the daughter scaffold severely disrupted, indicating that γ-tubulin is essential for its formation. The absence of a functional scaffold likely explains the observed “centriolar” SPMT phenotype (Fig. 5), visible after γ-tubulin depletion, as SPMTs presumably fail to transfer to the nAPR and remain associated with their nucleation place. Based on the time frame of experiments and the depletion system used, we can, however, not exclude that SPMT nucleation also depends on γ-tubulin.

While this study was in revision, Haase et al. reported similar phenotypic consequences of γ-tubulin disruption in *T. gondii* (70). The authors further demonstrated that the γ-tubulin small complex components GCP2 and 3 colocalize with γ-tubulin in *T. gondii*, and that both genes are essential for tachyzoite proliferation (70). Here, we dissected if and how the γ-TuRC components GCP4/5/6 act in microtubule nucleation by examining their localization patterns and phenotype upon depletion (Fig. 7). This showed that all three are essential and localize to the nAPR. Although their depletion does not mirror the γ-tubulin phenotype, it severely affects the efficiency of nSPMT raft assembly. What is curious, though, is that even if one nSPMT assembly is skipped, the SPMT grouping in rafts proceeds as if the nSPMT was assembled. As such, γ-TuRC is critical for correct nSPMT raft architecture and may facilitate specificity of γ-tubulin localization and/or function at the forming scaffold. We currently do not know how γ-tubulin is differentially targeted to a variety of structures but note that all of them are located closely together and are formed at the same time, suggesting differences in the composition of the respective γ-TuRCs. To gain the necessary fine-tuning, γ-TuRCs could, for example, rely on post-translational modifications, and GCP2 was recently identified as a substrate of TgCrk4, a kinase that acts upstream of the spindle assembly and centrosome reduplication checkpoint (71).

While depletion of γ-tubulin/γ-TuRC has severe effects on scaffold formation, it does not prevent the initiation of budding. As previously reported by Striepen and coworkers, our data confirm that this process depends on the presence of SFA2 and by extension on the SFA fiber (19). Notably, our short 2 h disruption of SFA2 does not result in aberrant daughter IMC3 signal (Fig. 8E) and formation of SPMTs connected to centrioles (Fig. 8F), as observed for our 2 h γ-tubulin depletion (Fig. 4 to 6; Fig S3C and D and 5A and C). This direct comparison suggests that the function of the SFA fiber precedes that of γ-tubulin/γ-TuRC. While our SFA2 depletion results in the formation of a single bud per mother cell, the previous SFA2 characterization has reported that budding is not initiated at all (19). We speculate that the fast-acting auxin-degron system used here depletes SFA2 beyond a critical threshold, permitting scaffold formation by only one centrosome.

Further work is therefore necessary to clarify how the SFA fiber is priming daughter bud formation. However, its previously hypothesized direct role in microtubule assembly (19) can now be attributed to γ-tubulin/γ-TuRC.

Here, we also explored the connection between the number of nSPMT rafts and the architecture of the alveolar plate organization in the IMC. TEM demonstrated the presence of membrane on top of the SPMT rafts (Fig. 2C), which is in line with a few other published TEM micrographs revealing the organization of the IMC around fivefold nSPMT raft symmetry (72, 73) and a more recent Cryo-ET study (57). The key observation is the discontinuity between the neighboring membrane sheets, which we infer is where the longitudinal sutures between the alveolar plates are positioned. We employed BCC0 and IMC32 as markers of this alveolar architecture, since both have a localization reminiscent of the daughter IMC sutures (21, 23). Depolymerizing nSPMT via oryzalin demonstrates that the symmetrical deposition of the longitudinal signal is lost, which puts nSPMT raft organization before putting down the IMC sutures. Furthermore, the 11 alveolar plates in *S. neurona* corresponded directly with nSPMT rafts composed of two nSPMTs (Fig. 3). Thus, across two different coccidian parasites, the deposition of nSPMT rafts cues the number of alveolar plates. Furthermore, there is a strong proclivity to form paired MTs observed in both *T. gondii* and *S. neurona*, as well as upon γ-tubulin depletion (Fig. 2B, 3C, and 4F). Whether these rules apply universally across all Apicomplexa is unlikely, since different “zoites” display vastly different alveolar quilts and SPMT numbers (2), but it at least applies to the coccidia.

A distinct feature in the coccidia is the presence of a single apical cap alveolus, separated from the central and basal alveolar plates. Upon depletion of γ-tubulin, we learned that initial positioning of the apical cap (as marked by membrane-anchored ISP1 [67]) is independent from nSPMTs, but that its subsequent formation as well as the assembly of the central and basal rows of alveoli does require the nSPMTs (Fig. S5; Fig. 6). This aligns with previous observations using oryzalin, demonstrating the nSPMTs are not required for ISP1 deposition (67). Moreover, we have recently shown that the basis for the IMC is the fivefold BCC0 organization, from which the apical cap is assembled in the apical direction, and the central and basal alveolar plates in the basal direction (Fig. 1A).

At the mid-budding stage, the nSPMT raft organization transitions into an equal distribution of nSPMTs around the APR. Comparison of *T. gondii* tachyzoites with *S. neurona* merozoites indicates this could be tailored to individual needs, since even in completely formed *S. neurona* merozoites the SPMTs still have a paired appearance (Fig. 3C). A recent study in *T. gondii* found that equal distribution of nSPMTs coincides with the alignment of APR1 to the APR (56). Although mechanistically, we currently have no insights into how the distribution or maintenance of clustering is organized. Another distinctive and putatively related feature is the differential length of the SPMTs: in *T. gondii,* they are largely of similar length running to about two-thirds the length of the tachyzoite, whereas in *S. neurona* merozoites, the pairs consist of one long MT running all the way to the basal complex, and one MT ending at about two-thirds the length of the merozoite (Fig. 3C). Clearly, there is very precise control of the SPMT length, which could be related to their raft organization.

Overall, the pairing of pan-ExM with genetic dissection of early SPMT formation and its interface with other cytoskeletal elements has delivered several advancing new insights regarding the interdependent assembly of the different apicomplexan cytoskeleton elements. We also uncovered a new correlation between SPMT and alveolar architecture. However, many questions remain, e.g., how the parasite counts SPMTs, and how they transition from the centrosome to the nAPR. Thus, the insights and tools developed here raised these new questions while at the same time providing the tools needed to answer them in future work.

## MATERIAL AND METHODS

### Parasites and host cell cultures

All *T. gondii* strains are derived from RH, and tachyzoite cultures were maintained in human telomerase reverse transcriptase-immortalized human foreskin fibroblasts (HFFs) as previously described (74). Immunofluorescence assays were performed using primary HFFs. The *Sarcocystis neurona* SN3ΔHXGPRT-G11 strain (kindly shared by Dr. Daniel Howe, University of Kentucky) was maintained and imaged in bovine turbinate (BT—ATCC CRL-1390) cell monolayers described in references 75, 76, supplemented with a chemically defined lipid concentrate at a 1:1,000 dilution according to manufacturer protocol (Gibco 11905031). *S. neurona* stably transfected with tub-TgIMC15-eYFP to visualize parasite IMC sutures (63) was generated under 10 µM pyrimethamine selection and cloned by limiting dilution (75). Conditional depletion lines were established by targeting the endogenous 3´ end of γ-tubulin (TGGT1_226870A + B), SFA2 (TGGT1_205670), GCP4 (TGGT1_209970), GCP5 (TGGT1_288890), and GCP6 (TGGT1_285780) with a mAID-5xV5-DHFR homologous repair construct, after CRISPR/Cas9-induced DNA double-strand break in the respective region (Fig. S8A). For CRISPR/Cas9-based 3′ end tagging with ALFA-tag-YFP, we generated a homologous repair template in which the short ALFA-tag sequence (77) was fused to YFP-HXGPRT. Selection for stable *T. gondii* transfectants was performed under 1 µM pyrimethamine, 25 mg/mL mycophenolic acid, and 50 mg/mL xanthine, and stable lines were cloned by limiting dilution (γ-tub-cKD, GCP4-cKD, γ-tub-cKD+ISP1-ALFA-tag-YFP, and GCP6-cKD+γ-tub-ALFA-tag-YFP). Integration into the target gene locus and disruption of the endogenous gene 3′ end was verified by diagnostic PCR (Fig. S8) with oligonucleotide primers listed in Table S1. Protein knockdowns using the mAID system were performed under 500 µM auxin (IAA in 100% ethanol; Sigma-Aldrich) (64).

### (Immuno)fluorescence microscopy

Intracellular parasites grown overnight in a six-well plate containing coverslips confluent with HFF cells were fixed with 100% methanol and blocked for 30 min with 2% bovine serum albumin (BSA) in 1× phosphate buffered saline (PBS). The primary antisera (IMC3 and *Hs*Centrin2 [21], 1:1,000; ISP1 [67] and TgSORTLR [78], 1:500), streptavidin-ALEXA488 (1:1,000), and secondary antisera (Thermo Fisher Scientific; goat anti-mouse ALEXA488 and goat anti-rabbit ALEXA594, 1:500) were diluted in blocking solution and applied for 30 min at room temperature (RT), followed by three 5 min washes in 1× PBS. DNA staining was performed using DAPI in the first wash after the last antibody.

### Iterative expansion microscopy (pan-ExM)

Pan-ExM of *T. gondii* tachyzoites and *S. neurona* schizonts/merozoites was achieved by following published protocols (53, 54). Briefly, tachyzoites were grown overnight in six-well dishes on confluent HFF cells, treated for indicated time frames with auxin (IAA), and fixed with 4% paraformaldehyde (PFA). *S. neurona* merozoites were seeded on confluent BT cells and grown up to 120 h before fixation with 4% PFA in PBS. Samples were incubated in anchoring solution (0.7% formaldehyde, 1% acrylamide in PBS) for 3**–**4 h at 37°C. To achieve a consistent primary gel thickness, samples were placed in gelation chambers (53, 79) and incubated with primary gel solution (19% sodium acrylate, 10% acrylamide, 0.1% N,N′-(1,2-dihydroxyethylene)bisacrylamide (DHEBA) in PBS, 0.25% tetramethylethylenediamine (TEMED) and ammonium persulfate (APS) for 1 h at 37°C. Resulting gels were first denaturized in a buffer (200 mM SDS, 200 mM NaCl, 50 mM Tris, pH 6.8) for 15 min at RT and subsequently incubated at 73°C for 1 h. Gels were washed in PBS and left for the first expansion overnight in ultrapure water. The next day, a 2 × 2 cm gel piece was taken for further processing and incubated in neutral hydrogel solution (10% acrylamide, 0.05% DHEBA, in H_2_O, 0.05% TEMED and APS), twice—each time for 20 min—at RT. Excess hydrogel solution was carefully removed with a Kim Wipe before gels were sandwiched between a microscopy slide and a 22 mm coverslip, followed by a 1 h incubation at 37°C. Gels were then briefly washed in PBS and incubated twice—each time for 15 min on ice—in secondary gelation solution (19% sodium acrylate, 10% acrylamide, 0.1% bis-acrylamide in PBS, 0.05% TEMED and APS). Excess gelation solution was carefully removed with a Kim Wipe, gels were sandwiched between a microscopy slide and a 22 mm coverslip, and incubated for 1 h at 37°C. To dissolve DHEBA, gels were placed in 0.2 M NaOH in H_2_O for 1 h at RT and subsequently washed in PBS until the pH was neutral.

Pieces of the final gel were used for antibody staining. Primary antibodies (rabbit anti-V5 [Abcam], 1:300; rabbit anti-MORN1 [31], 1:500; rabbit anti-PolyE [AdipoGen], 1:500; rabbit anti-IMC6 [65], 1:500; mouse anti-α-tubulin [clone 12G10, Developmental Studies Hybridoma Bank {DSHB}], 1:250; rabbit anti-GFP [Torrey Pines Biolabs], 1:300; and guinea pig anti-ALFA-tag [N1584, Synaptic Systems, 1:400]) were diluted in 2% BSA in PBS and gels incubated overnight at RT, followed by a 3 h incubation step at 37°C the next day. Gels were then extensively washed in 0.1% PBST before being incubated in secondary antibody solution (Thermo Fisher Scientific; goat anti-mouse DyLight488, goat anti-rabbit DyLight594, goat anti-mouse ALEXA488, goat anti-rabbit ALEXA594, and goat anti-guinea pig ALEXA488, all 1:200). Incubation times and temperatures mirrored conditions used for primary antibodies and gels were washed in 0.1% PBST. To stain for protein density, gels were incubated in NHS-ester-ATTO488 (20 µg/mL in PBS) for 1 h and subsequently washed with 0.1% PBST. The final expansion was done in ultrapure water overnight. For imaging, expanded gels were placed in 35 mm glass-bottom dishes (Ibidi) coated with poly-L-lysine and imaged on a Zeiss LSM 880 microscope with Airyscan unit. Images were acquired with Zeiss Zen Black software, while Zeiss Zen Blue version 2.3 was used for image processing using standard settings. Final image processing was done in FIJI (80) by adjusting brightness and contrast.

### Single expansion of *Sarcocystis neurona* samples

Single expansion of *Sarcocystis* samples was done similar to a previously reported protocol (56). Briefly, the pan-ExM protocol was followed until the first expansion in ultrapure water overnight. Pieces of the gel were then incubated in PBS to allow gel shrinkage and subsequently stained with primary antibodies in 2% BSA/PBS overnight. Gels were washed in 0.1% PBST and subjected to secondary antibody staining overnight. Antibody dilutions were the same as used for pan-ExM. The next day, gels were extensively washed with 0.1% PBST. To stain for protein density, gels were incubated in NHS-ester-ATTO488 (5 µg/mL in PBS) for 1 h and subsequently washed with 0.1% PBST. The final expansion was done in ultrapure water overnight and gels were imaged the next day as stated in the pan-ExM section.

### TEM

Wild-type RH parasites were grown overnight in a T25 tissue culture flask confluent with HFF cells. The intracellular parasites were collected by trypsinizing the monolayer and fixed with 2.5% glutaraldehyde in 0.1 M phosphate buffer, pH 7.2. The samples were fixed in OsO_4_, dehydrated in ethanol, treated with propylene oxide, and embedded in Spurr’s epoxy resin. Sections were stained with uranyl acetate and lead citrate prior to examination in a JEOL 1200 EX electron microscope (81).

### Chemical perturbation of MT with oryzalin

Oryzalin treatment was done similar to previous reports (58, 59)***.** T. gondii* tachyzoites expressing endogenously tagged IMC32-5xV5 were grown in six-well plates, containing coverslips with confluent HFF cells, overnight. The next day, parasites were treated with a final concentration of 2.5 µM oryzalin in dimethyl sulfoxide and incubated for 2 h under standard culture conditions. Untreated IMC32-5xV5 expressing parasites were used as controls. Parasites were fixed with 4% PFA in PBS and subjected to the pan-ExM protocol.

### Western blotting

For western blotting, lysates of 2 × 10^6^ parasites (either IAA treated/not treated γ-tub-cKD, SFA2-cKD, GCP4-cKD, GCP5-cKD, GCP6-cKD, GCP6-cKD+γ-tub-ALFA-tag-YFP or RH∆Ku80-Tir1 parasites) were loaded on/separated via SDS-PAGE and proteins transferred on polyvinylidene difluoride membranes. Membranes were blocked in 6% milk in PBS and subsequently stained with primary mouse anti-V5 (clone SV5-Pk1, BioRad, 1:10,000) or rabbit anti-ALFA-tag (N1583, Synaptic Systems, 1:10,000) antibodies. The secondary antibodies (goat anti-mouse or goat anti-rabbit, 1:100,000) are coupled to horseradish peroxidase (HRP), and the Super Signal West Atto Ultimate Sensitivity Substrate (Thermo Scientific) was used as a HRP substrate. The membrane was stripped of antibodies, and mouse anti-α-tubulin (12G10, DSHB, 1:15,000) or mouse anti-IMC1 (1:10,000/1:15,000) antibodies were used as loading controls.

## SUPPLEMENTAL MATERIAL

Supplemental Material mbio.01389-25-s0001.pdf Fig. S1 to S8.

Table S1 mbio.01389-25-s0002.xlsx Oligonucleotide primers, in 5' to 3' orientation.

ASM does not own the copyrights to Supplemental Material that may be linked to, or accessed through, an article. The authors have granted ASM a non-exclusive, world-wide license to publish the Supplemental Material files. Please contact the corresponding author directly for reuse.
