# Supplementary material for: Co-dependent formation of the Toxoplasma gondii subpellicular microtubules and inner membrane skeleton
Source: mBio. 2025 Aug 13;16(9):e01389-25. doi: 10.1128/mbio.01389-25 (PMC12421850; doi:10.1128/mbio.01389-25)
Supplement: Supplemental Material — Fig. S1 to S8. [file mbio.01389-25-s0001.pdf]

Supplemental Material

Co-dependent formation of the *Toxoplasma gondii* subpellicular microtubules and inner membrane skeleton

Klemens Engelberg, Ciara Bauwens, David J. P. Ferguson and Marc-Jan Gubbels

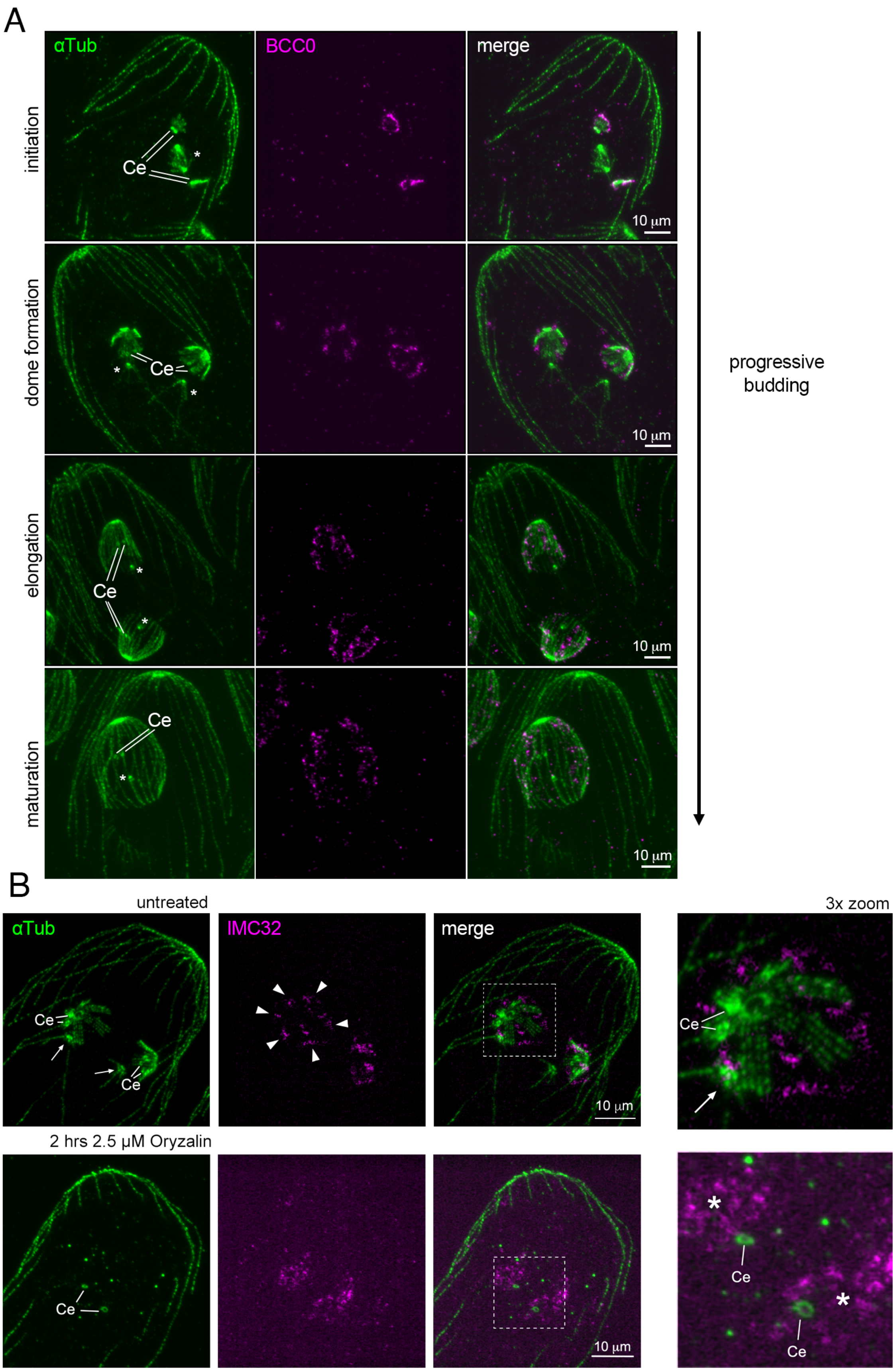
A
αTub
BCC0
merge
initiation
dome formation
elongation
maturation
Ce
*
10 µm
progressive
budding
B
untreated
IMC32
3x zoom
2 hrs 2.5 µM Oryzalin

Figure S1: A. Pan-ExM of BCC0-5xV5-expressing parasites during distinct steps of endodyogeny. BCC0 is already present when budding is initiated, surrounding the forming daughter scaffolds. With ongoing bud growth, BCC0 relocalizes to the space between nSPMTs rafts, and appears in five longitudinal stripes, reminiscent of the daughter IMC sutures, for the subsequent bud elongation and maturation steps. **B.** Pan-ExM of IMC32-5xV5-expressing parasites treated ± 2.5 μM Oryzalin for 2 hrs. As previously observed [1, 2] division is not halted by this treatment, but cytoskeleton organization is substantially altered. After Oryzalin treatment, IMC32-5xV5 forms plaques (asterisks) around (single) centrioles, contrary to its localization in discrete foci (arrowheads) between nSPMT rafts in untreated controls. Note that the scaffold in this image exhibits a six-fold symmetry, which is sometimes observed [3], although the five-fold arrangement appears to be the most abundant form [4]. Ce: centrioles; arrows: mitotic spindle. αTub: α-tubulin antiserum (mAb 12G10).

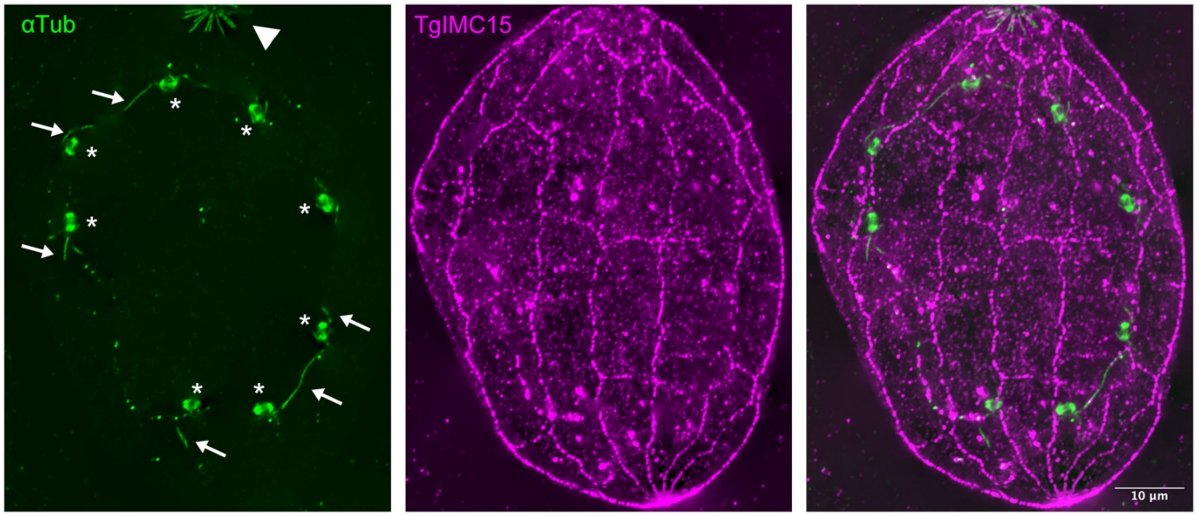

Figure S2: Image depicts a wider z-range than shown in Figure 3B. Arrowhead highlights the apical end with SPMTs, asterisks the mitotic spindle and arrows indicate individual microtubules associated with the mitotic spindle. αTub: α-tubulin antiserum (mAb 12G10).

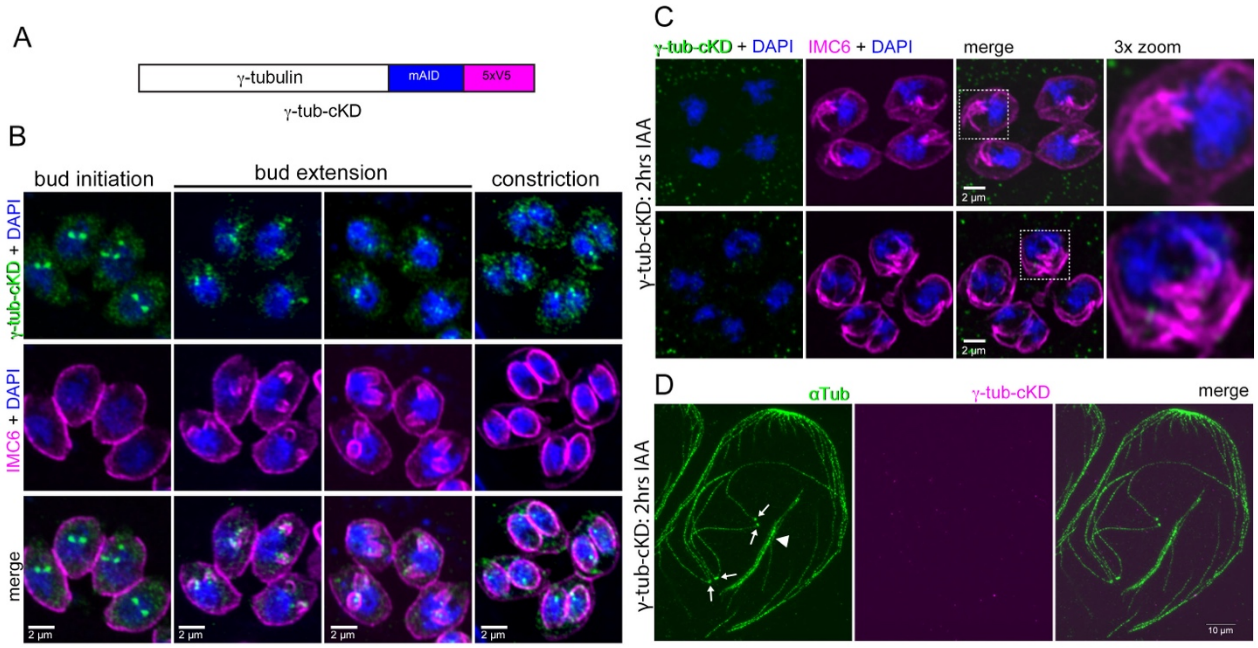

Figure S3: A. Schematic of the endogenously expressed γ-tubulin fusion (γ-tub-cKD), tagged at the 3'end with mAID and 5xV5. **B.** Immunofluorescence assay of γ-tub-cKD parasites, co-stained with anti-IMC3 antiserum. Anti-V5 antiserum recognizes γ-tub-cKD, DAPI stains DNA. **C.** Immunofluorescence assay of γ-tub-cKD parasites, co-stained with anti-IMC3 antiserum. Parasites were treated with 500 µM IAA for 2 hr before fixation. Degradation of γ-tub-cKD is confirmed by use of anti-V5 antiserum and absence of specific staining in the γ-tub-cKD panel. Zoom images show unstructured IMC3 signal. **D.** pan-ExM of parasites expressing γ-tub-KD, treated for 2 hr with 500 µM IAA before fixation. Degradation of γ-tub-cKD is confirmed by use of anti-V5 antiserum and absence of specific staining in the γ-tub-cKD panel. Arrows indicate round structures, observed at the end of a microtubule population, arrowhead indicates bundle of microtubules. αTub: α-tubulin antiserum (mAb 12G10).

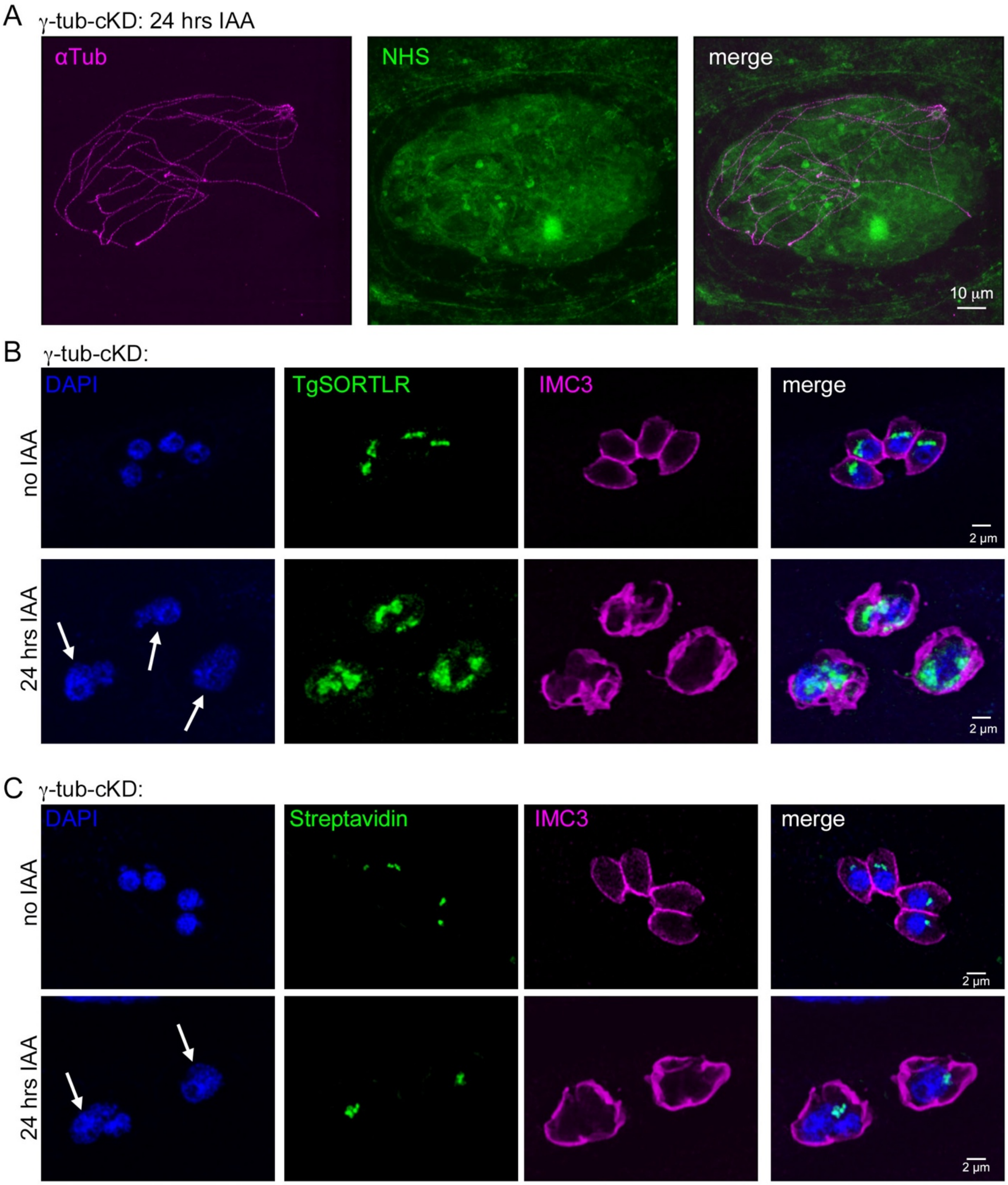

Figure S4: A. Depletion of γ-tubulin for 24 hr imaged by pan-ExM reveals long unstructured MTs without visible daughter cytoskeletons in the cytosol of an unstructured mother cell. NHS: NHS-ATTO488 highlights protein density; αTub: α-tubulin antiserum mAb 12G10. **B.** Use of TgSORTLR antiserum highlights Golgi-endosomal related compartments in γ-tub-cKD parasites treated ± IAA for 24 hrs. In IAA-treated parasites the compartments increase substantially in size. The nuclei (arrows) also increase in size and fail to segregate to the extent seen in controls. **C.** Streptavidin,

fused to ALEXA488, recognizes endogenously biotinylated proteins in the apicoplast. Upon IAA treatment of γ-tub-cKD, the apicoplast fails to segregate. IMC3 antiserum in B&C highlights the IMC and DAPI binds to DNA. Arrows: enlarged nuclei.

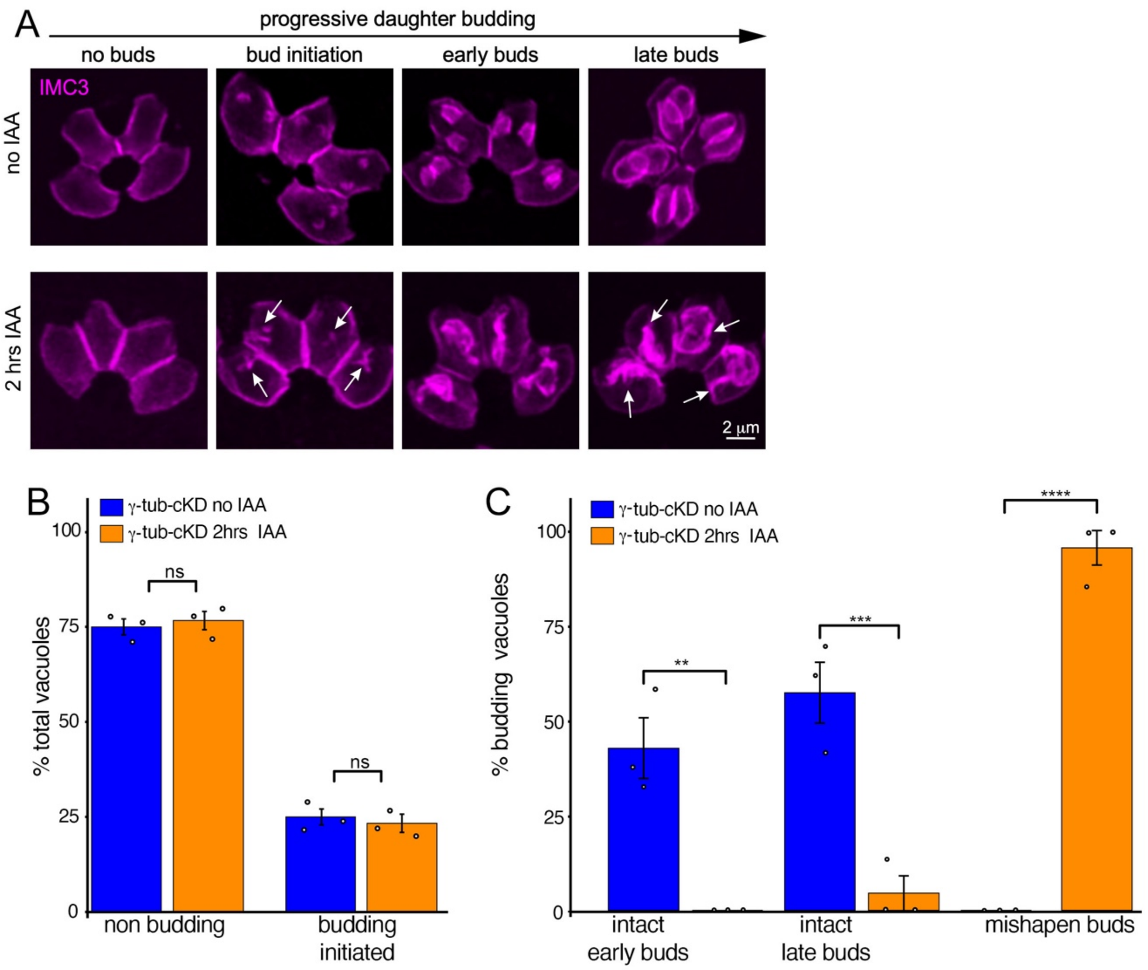

Figure S5: A. Airyscan superresolution images of 2 hr γ-tubulin depleted parasites. No interference with initiation of daughter budding is observed (arrows in ‘bud initiation’ panel) but the morphology of the daughter buds is severely affected: daughters appear disrupted, and no dome-shape bud is assembled when γ-tubulin is depleted (arrows in ‘late buds’ panel).

B. Quantification of parasites that initiated budding, judged by presence of daughter IMC3 signal after 2 hr of γ-tubulin depletion. n=3 biological replicates, 100 vacuoles each. **C.** Quantification of daughter bud integrity based on budding vacuoles quantified under F.

For B&C: Error bars denote SEM. Data points indicate individual replicates. One-way ANOVA with Tukey’s HSD. ns: not significant; **: $p < 0.01$; ***: $p < 0.001$; ****: $p < 0.0001$.

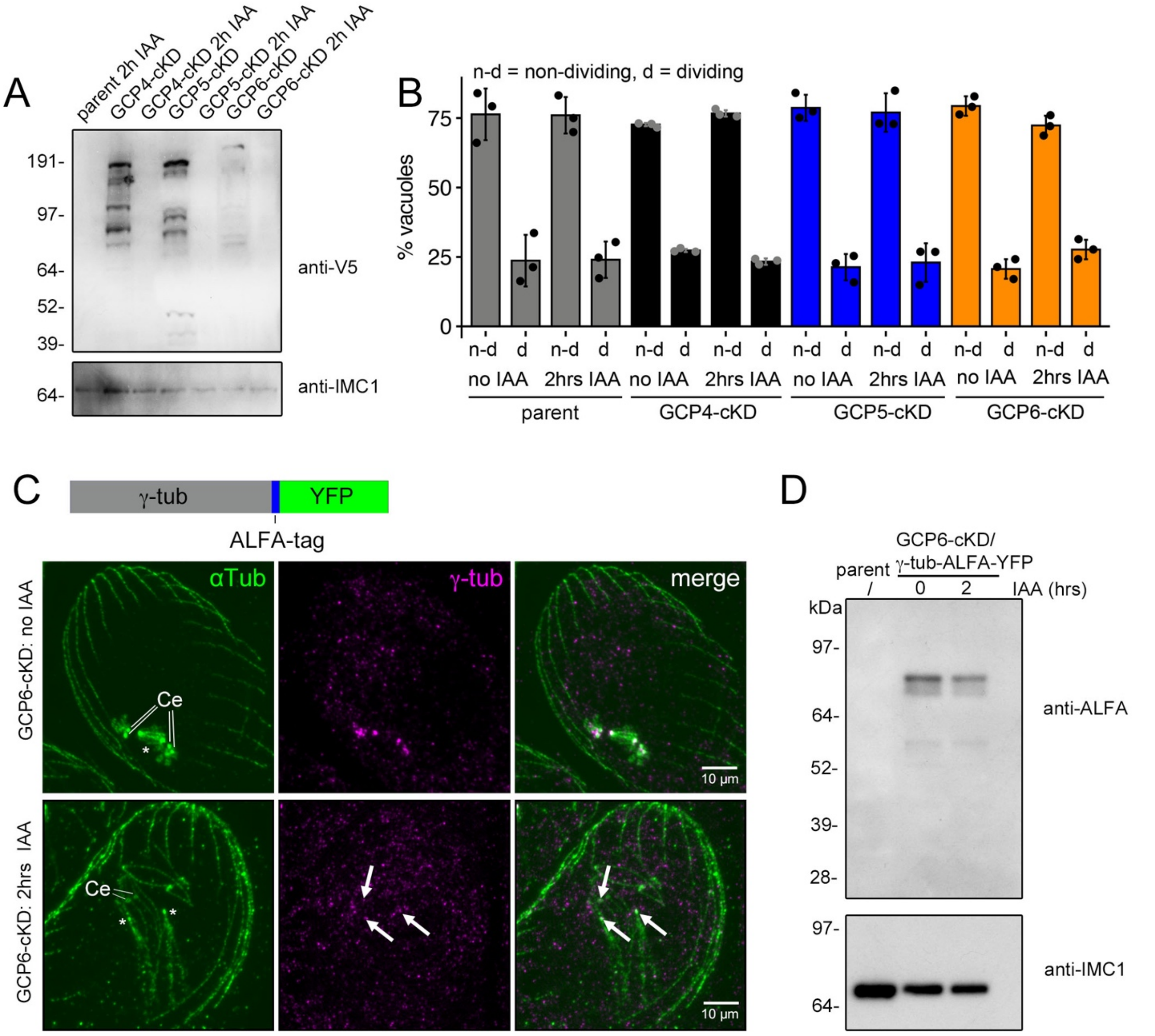

Figure S6: A. Western blot analyses of GCP-cKD proteins upon 2 hr IAA treatment. GCP4-6 cKD were detected with V5 antiserum, IMC1 antiserum was used as loading control. Parent: RHΔKu80-Tir1. **B.** Quantification of vacuoles harboring non-dividing vs. dividing parasites for GCP4/5/6-cKD compared to parent (RHΔKu80-Tir1). No significant differences between the number of vacuoles harboring non-dividing or the number of dividing parasites were found, irrespective of the condition. 100 vacuoles were quantified for each of the n = three biological replicates. One-way ANOVA with Tukey's HSD was used to test for significance. Error bars indicate SD. **C.** Pan-ExM of GCP6-cKD parasites expressing γ-tubulin endogenously tagged with AFLA-tag YFP. Upon 2 hr IAA treatment, γ-tubulin loses its localization to the forming scaffold, and shows decreased abundance at the centrioles (Ce) and spindle poles (asterisks). Arrows highlight remaining localization of γ-tubulin. γ-tubulin was detected with GFP antiserum; αTub: α-tubulin antiserum

mAb12G10. **D.** Western blot analyses of endogenously tagged γ-tubulin after 2 hrs IAA treatment in the GCP6-cKD background. ALFA-tag antiserum recognizes γ-tubulin, IMC1 antiserum was used as loading control. Parent: RHΔKu80-Tir1.

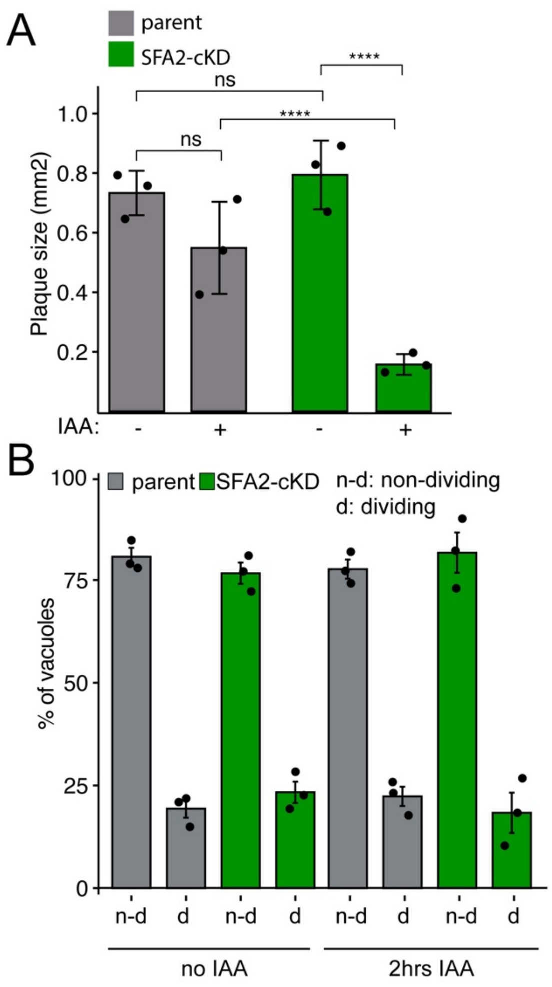

Figure S7: A. Plaque size measurement of three biological replicates (individual dots) for the parent (RHΔKu80-Tir1) or SFA2-cKD parasites. The size of 20 plaques were measured for each condition, after a seven-day incubation ± IAA. One-way ANOVA with Tukey's HSD was used to test for significance. Error bars indicate SD. Ns: not significant; ****: $p < 0.0001$. **B.** Quantification of vacuoles harboring non-dividing vs. dividing parasites for SFA2-cKD compared to RHΔKu80-Tir1. No significant differences between the number of vacuoles harboring non-dividing or the number of dividing parasites were found, irrespective of the condition. 100 vacuoles were quantified for each of the n = three biological replicates. One-way ANOVA with Tukey's HSD was used to test for significance. Error bars indicate SEM.

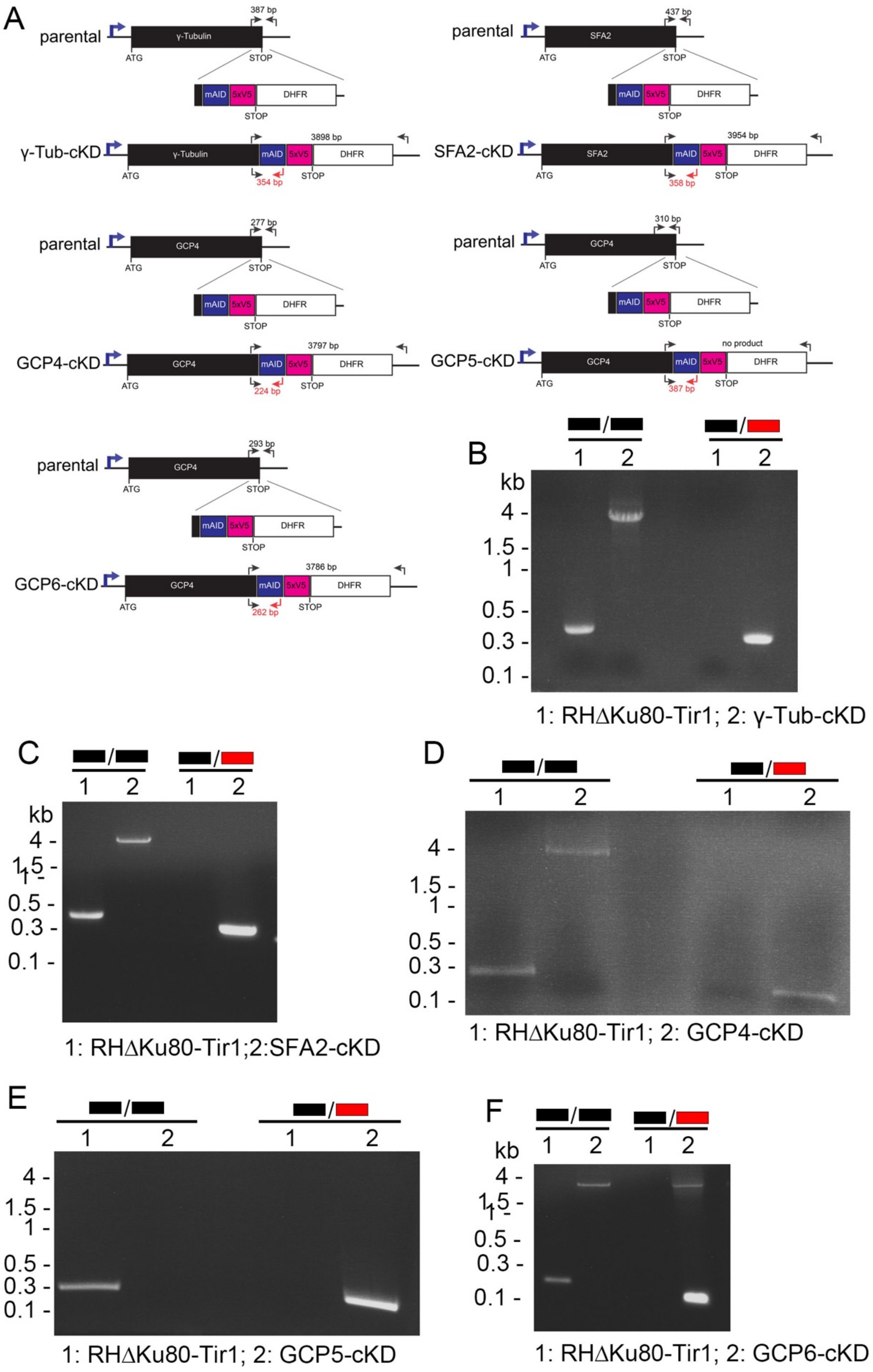
A
parental
γ-Tubulin
387 bp
ATG
STOP
mAID
5xV5
DHFR
γ-Tub-cKD
3898 bp
354 bp
SFA2
437 bp
SFA2-cKD
3954 bp
358 bp
GCP4
277 bp
GCP4-cKD
3797 bp
224 bp
310 bp
GCP5-cKD
no product
387 bp
293 bp
GCP6-cKD
3786 bp
262 bp
B
kb
1: RHΔKu80-Tir1; 2: γ-Tub-cKD
C
1: RHΔKu80-Tir1;2:SFA2-cKD
D
1: RHΔKu80-Tir1; 2: GCP4-cKD
E
1: RHΔKu80-Tir1; 2: GCP5-cKD
F
1: RHΔKu80-Tir1; 2: GCP6-cKD

Figure S8: A. Schematic of the endogenous gene locus of γ-tubulin, SFA2, GCP4, GCP5 and GCP6 and introduced modifications. DNA double strand break at the 3'end of the respective gene was induced via CRISPR/Cas9 and a homologues repair fragment, including the mAID and 5xV5 coding sequence, co-transfected. Expected fragment size for diagnostic PCRs are indicated in the schematics of each respective gene.

B. Diagnostic PCR for γ-tub-cKD.

C. Diagnostic PCR for SFA2-cKD.

D. Diagnostic PCR for GCP4-cKD.

E. Diagnostic PCR for GCP5-cKD.

F. Diagnostic PCR for GCP6-cKD. RHΔKu80-Tir1 parasites served as a control in all PCRs. kb indicates kilo bases.

Supplemental References

1. Stokkermans, T.J., et al., *Inhibition of Toxoplasma gondii replication by dinitroaniline herbicides.* Exp Parasitol, 1996. **84**(3): p. 355-70.
2. Morrissette, N.S. and L.D. Sibley, *Disruption of microtubules uncouples budding and nuclear division in Toxoplasma gondii.* J Cell Sci, 2002. **115**(Pt 5): p. 1017-25.
3. Li, Z., et al., *Cryo-Electron Tomography of Toxoplasma gondii Indicates That the Conoid Fiber May Be Derived from Microtubules.* Adv Sci (Weinh), 2023. **10**(14): p. e2206595.
4. Padilla, L.F.A., J.M. Murray, and K. Hu, *The initiation and early development of the tubulin-containing cytoskeleton in the human parasite Toxoplasma gondii.* Mol Biol Cell, 2024: p. mbcE23110418.
